# FE-ToolKit: A Versatile Software Suite for Analysis of High-Dimensional Free Energy Surfaces and Alchemical Free Energy Networks

**DOI:** 10.1021/acs.jcim.5c00554

**Published:** 2025-05-21

**Authors:** Timothy J. Giese, Ryan Snyder, Zeke Piskulich, German P. Barletta, Shi Zhang, Erika McCarthy, Şölen Ekesan, Darrin M. York

**Affiliations:** Laboratory for Biomolecular Simulation Research, Institute for Quantitative Biomedicine and Department of Chemistry and Chemical Biology, 242612 Rutgers University, Piscataway, New Jersey 08854, United States

## Abstract

Free energy simulations play a pivotal role in diverse biological applications, including enzyme design, drug discovery, and biomolecular engineering. The characterization of high-dimensional free energy surfaces underlying complex enzymatic mechanisms necessitates extensive sampling through umbrella sampling or string method simulations. Accurate ranking of target-binding free energies across large ligand libraries relies on comprehensive alchemical free energy calculations organized into thermodynamic networks. The predictive accuracy of these methods hinges on robust, scalable tools for networkwide data analysis and extraction of physical properties from heterogeneous simulation data. Here, we introduce FE-ToolKit, a versatile software suite for the automated analysis of free energy surfaces, minimum free energy paths, and alchemical free energy networks (thermodynamic graphs).

## Graphical abstract

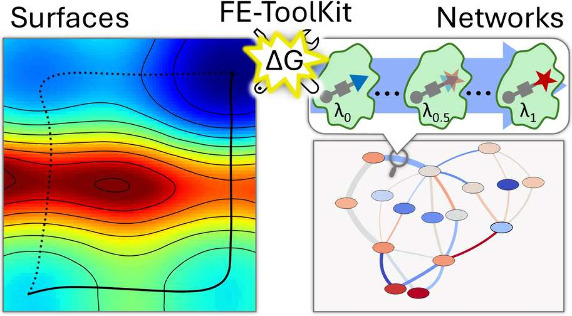

## Introduction

The FE-ToolKit software is used to analyze and visualize high-dimensional free energy surfaces and alchemical free energy networks. FE-ToolKit consists of 3 main components: ndfes, edgembar, and fetkutils. The ndfes component analyzes umbrella sampling to produce multidimensional free energy surfaces^1^ (FES) and optimize minimum free energy paths using the surface accelerated string method.^2^ The edgembar component analyzes alchemical free energy (AFE) simulations^3^ to calculate relative free energies between reference and target environments, e.g., in binding or solvation processes. The relative free energy simulations can be collected to form a topological network of transformations (sometimes referred to as a thermodynamic graph), and edgembar will perform networkwide free energy analysis to enforce cycle closure conditions and (optionally) additional experimental constraints.^4^ The fetkutils component contains programs to choose optimized AFE λ-schedules.^5^ Also contained in fetkutils are utilities to calculate kinetic isotope effects from umbrella sampling and path integral molecular dynamics (PIMD) simulations.^6^ These tools have been described in detail elsewhere.^7^

Free energy applications analyze a large number of simulations. High-dimensional free energy surfaces often use data from many umbrella sampling simulations (sometimes several thousand^8^). AFE networks consist of multiple edges (transformations) composed of alchemical “*λ* states” simulated within several independent trials to obtain averages and error estimates. Specialized methods and algorithms are required to efficiently perform global FES^1^ or networkwide AFE^4^ analysis. In addition, one quickly becomes burdened with managing and examining hundreds (or thousands) of simulations to identify unequilibrated sampling, poor phase space overlap with neighboring states, data correlation, and statistical outliers. An automated process is necessary to detect problematic sampling and to summarize a wide array of potential issues for the user. The FE-ToolKit software includes algorithms for automatically detecting and discarding unequilibrated sampling. In addition, FE-ToolKit reports a wide array of indexes that can be used to alert the user to potential problems and analyses to facilitate troubleshooting. The details of these algorithms, a description of the error analysis, and an extended discussion of the theory are provided in the Supporting Information.

In summary, FE-ToolKit (Figure 1) provides the following features and capabilities:

- Multistate Bennett Acceptance Ratio^9^ (MBAR) and/or variational free energy profile^1,10,11^ (vFEP) analysis of high-dimensional free energy surfaces.
- Determination of minimum free energy paths using the surface accelerated string method.^2^
- Networkwide analysis of thermodynamic graphs with Lagrange multiplier constraints for cycle closure conditions and experimental priors.^4,12,13^

- Interoperability with equilibrium and nonequilibrium work simulations,^14–16^ as well as indirect end state “book-ending” free energy corrections.^17^
- Automated determination of equilibrated sampling regions and outlier trial detection.
- Robust error analysis that considers correlation of time series data and independent trials, as well as cycle closure conditions.
- Trouble-shooting analysis, including calculation of Lagrange multiplier indexes, d*U*/dλ profiles and variances, phase space overlap, and replica exchange efficiency.
- Tools for determination of optimized λ schedules using phase space overlap, Kullback–Leibler divergence and replica-exchange acceptance ratio methods.^5^

The Amber/AmberTools^18,19^ software can perform GPU-accelerated alchemical free energy simulations with molecular mechanics force fields^12,13,20–22^ and umbrella sampling simulations using generalized quantum mechanical/molecular mechanical and machine learning potentials.^23,24^ This includes recently developed range-corrected deep-learning potentials,^25,26^ graph neural network potentials,^27^ and the QDπ models developed for drug discovery applications.^28,29^ The FE-ToolKit package has been integrated into Amber-specific free energy workflows;^30^ however, it reads data through its own input file formats rather than directly parsing simulation output. In this manner, the analysis programs are independent of the simulation package. The file formats are described here, the Supporting Information, and the Quick Start Tutorial.^31^ The input, output, and command-line options of all software within FE-ToolKit use kcal/mol energy units unless explicitly overridden by the user.

**Figure 1:**
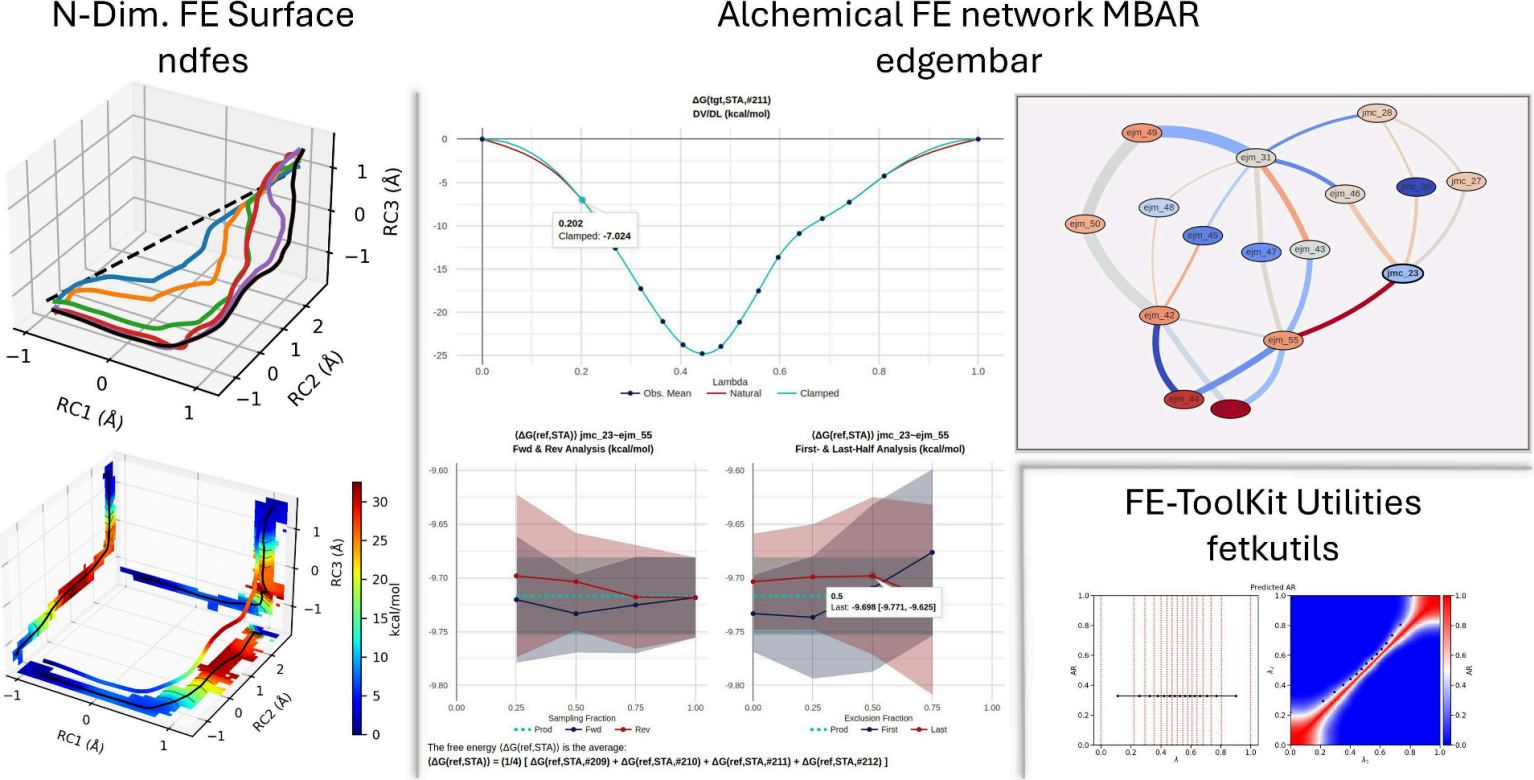
FE-ToolKit consists of ndfes for calculating *N*-dimensional free energy surfaces, edgembar for analyzing alchemical free energy networks using the EdgeMBAR method, and FE-ToolKit utilities (fetkutils) for optimizing schedules of alchemical states.

## Umbrella Sampling Free Energy Analysis

Umbrella sampling is used to study reaction mechanisms by introducing bias functions (harmonic potentials) to enhance the probability of observing rare configurations, such as transition states.^32–35^ An unbiased FES is obtained with biased sampling performed along relevant reaction coordinate values using either the MBAR^9^ or vFEP method.^4,10,11^ Other analysis techniques include umbrella integration,^36–38^ weighted histogram analysis^39,40^ (WHAM), and unbinned WHAM^41,42^ (UWHAM). The reweighting procedure can be extended to predict the FES of a target potential energy function from biased sampling obtained with inexpensive reference potentials. The weighted thermodynamic perturbation^43,44^ (wTP) and generalized weighted thermodynamic perturbation^45^ (gwTP) methods predict the high-level surface from sampling produced by one or more reference potentials, respectively.

The ndfes program produces multidimensional FESs by using the vFEP, MBAR, wTP, and gwTP methods. The input is a “metafile” whose lines describe the biased states. It is a generalization of the input used by Alan Grossfield’s WHAM program.^46^ Each state is characterized by an integer index (“Hamiltonian index”) that denotes the unbiased potential energy function, the harmonic force constants and positions used during sampling, and the simulation temperature. Each line of the metafile also provides a “dumpave” filename whose rows are the observed samples and whose columns are the simulation time and the reaction coordinate values. Additional columns of unbiased potential energies of the reference and target potentials are included if a wTP or gwTP analysis is desired. The Hamiltonian index within the metafile indicates which of the extra columns corresponds to the sampled state’s unbiased potential energy. The ndfes output is an Extensible Markup Language (XML) file that defines a multidimensional histogram and includes information for each occupied bin: the free energy value, standard error, the number of samples, and the reweighting entropy.^47^ The ndfes input and output formats are independent of the molecular dynamics software used to generate the biased sampling; however, the ndfes-PrepareAmberData.py script is provided as a convenience to help create metafile and dumpave files from simulations performed with the sander software. Examples of the ndfes metafile and dumpave formats are found in the Supporting Information and Quick Start Tutorial.^31^

FE-ToolKit is packaged with utilities to prepare, analyze, and visualize FESs. The ndfes-CombineMetafiles.py script combines multiple metafiles to analyze aggregate sampling drawn from multiple trials. The ndfes-AvgFESs.py script reads multiple FESs and outputs an average FES. The FE-ToolKit package includes examples that illustrate 2- and 3-dimensional FESs using the ndfes companion python library. The ndfes-CheckEquil.py utility uses the biasing potential time series to identify unequilibrated sampling within a dumpave file.

The ndfes-path program included within FE-ToolKit implements the surface-accelerate string method^2^ (SASM) and the modified string method in collective variables.^48^ The SASM method differs from other string methods^48–53^ by propagating the string from the aggregate sampling produced from all previous iterations using fast methods for robust evaluation of high-dimensional free energy surfaces.^1^ The available sampling is analyzed to produce a best estimate of the FES, and the current estimate of the minimum free energy path is optimized on the fixed surface.

The ndfes-genbias program is similar to ndfes; however, it does not assume that the simulations are biased with harmonic potentials. Instead, the values of the biasing potentials are read from extra columns within the dumpave files. The ndfes-genbias metafile format does not include umbrella window positions and force constants; it provides a “bias index” to indicate which of the extra columns corresponds to the bias used during sampling. Further details regarding the input format can be found in the Supporting Information. We recommend using ndfes rather than ndfes-genbias whenever possible. The ndfes input files and memory requirements are much smaller because it computes the bias potential as needed. The ndfes-genbias program is not yet capable of performing the vFEP method. Finally, one must exercise caution when aggregating the samples obtained from multiple trials and reference potentials because the “bias indexes” are invalidated if the metafiles do not use the same ordered set of biasing potentials. Similarly, all ndfes-genbias dumpave files would need to be completely rewritten if a new biasing potential was encountered.

## Alchemical Free Energy Analysis

The edgembar program analyzes networks (graphs) of AFE simulations where the nodes and edges represent ligands and alchemical transformations, respectively. The free energy of an edge connecting ligands *a* and *b* is decomposed into contributions from two environments ΔΔ*G*_(*ab*)_ = Δ*G*_(*ab*),target_ – Δ*G*_(*ab*),ref_. The transformation in an environment is decomposed into stages, Δ*G*_(*ab*)*e*_ = ∑_*s*_Δ*G*_(*ab*)*es*_, using either a one-stage softcore–electrostatic^54–56^ or three-stage split protocol.^57^ The free energy of a stage is an average of multiple independent simulation trials, Δ*G*_(*ab*)*es*_ = ⟨Δ*G*_(*ab*)*est*_⟩, where *t* indexes the trial. A “trial” is a set of simulations performed at *N*_state,(*ab*)*est*_ states spanning λ ∈ [0, 1], which define the potential energy, *U*_(*ab*)*es*_(**r**;λ). The trial’s free energy is the difference between its final and initial states, Δ*G*_(*ab*)*est*_ = *G*_(*ab*)*est*,λ=1_ – *G*_(*ab*)*est*,λ=0_. MBAR analysis of trial *t* is equivalent to minimization of a convex objective function,^42^ *F*_(*ab*)*est*_(**G**), with respect to *N*_state,(*ab*)*est*_ state free energies, **G**_(*ab*)*est*_.

$$F_{\left(ab\right)est}\left(G_{\left(ab\right)est}\right)=\frac{1}{N_{s,\left(ab\right)est}}\overset{N_{\mathrm{state},\left(ab\right)est}}{\underset{j=1}{\sum }}\overset{N_{s,\left(ab\right)estj}}{\underset{k=1}{\sum }}\times \ln\left(\overset{N_{\mathrm{state},\left(ab\right)est}}{\underset{l=1}{\sum }}\exp\left[-\beta U_{\left(ab\right)es}\left(r_{\left(ab\right)estjk};\lambda_{l}\right)-b_{\left(ab\right)estl}\right]\right)+\overset{N_{\mathrm{state},\left(ab\right)est}}{\underset{i=1}{\sum }}\frac{N_{s,\left(ab\right)esti}}{N_{s,\left(ab\right)est}}b_{\left(ab\right)esti}$$ 1

Here, β = 1/*k*_B_ *T*, where *k*_B_ is the Boltzmann’s constant, *T* is the absolute temperature, *N*_s,(*ab*)*esti*_ is the number of samples drawn from state *i*, *N*_s,(*ab*)*est*_ is the aggregate number of samples within trial *t*, **r**_(*ab*)*estjk*_ is sample *k* in the ensemble of state *j* from trial *t*, and *b*_(*ab*)*esti*_ is shown in eq 2.

$$b_{\left(ab\right)esti}=-\ln\frac{N_{s,\left(ab\right)esti}}{N_{s,\left(ab\right)est}}-\beta G_{\left(ab\right)esti}$$ 2

Alternatively, one can define an objective function for the entire edge, *F*_(*ab*)_(**G**_(*ab*)_), and simultaneously solve for every state in each environment, stage, and trial.

$$F_{\left(ab\right)}\left(G_{\left(ab\right)}\right)=\underset{e}{\sum }\overset{N_{\mathrm{stage}}}{\underset{s=1}{\sum }}\frac{\overset{N_{\mathrm{trial},\left(ab\right)es}}{\underset{t=1}{\sum }}F_{\left(ab\right)est}\left(G_{\left(ab\right)est}\right)}{N_{\mathrm{trial},\left(ab\right)es}}$$ 3

The edge free energy is calculated from these values, ΔΔ*G*_(*ab*)_(**G**_(*ab*)_^*^), where the asterisk denotes the energies which minimize eq 3.

The sum of edge free energies along any closed path in the network should be zero; however, this is not guaranteed when the edges are independently analyzed. To rectify this, the MBARnet method^4^ calculates every state in the network by minimizing a graph objective function (a sum of edge objectives) while imposing constraints to enforce closure conditions on minimal length cycles (cycles that cannot be formed by the union of smaller cycles). The MBARnet method has several shortcomings. The graph objective is expensive to evaluate; it requires a large amount of computer memory; the optimization needs to be performed if any new data or edges are added or removed; and enforcement of minimal length cycle closures does not guarantee that larger cycles will close.

The EdgeMBAR method avoids these shortcomings by introducing a graph objective function expressed in terms of *N*_lig_ – 1 ligand free energies. One ligand defines the arbitrary zero of energy, and the remaining free energies are relative to the reference *c*_*a*_ = Δ*G*_*a*_ – Δ*G*_0_. The graph objective function is a sum of effective edge objectives *F*(**c**) = ∑_(*ab*)_ *F̃*_(*ab*)_(*c*_*b*_ – *c*_*a*_). The argument of an effective edge objective is a scalar value: the edge free energy. Values of the edge objective function can be pretabulated from constrained optimizations.

$$\begin{array}{rll} {\mathrm{F̃}}_{\left(ab\right)}\left(x\right)= & \underset{G_{\left(ab\right)}}{\min}F_{\left(ab\right)}\left(G_{\left(ab\right)}\right) & \\ & \mathrm{subject to}:\Delta \Delta G_{\left(ab\right)}\left(G_{\left(ab\right)}\right)=x \end{array}$$ 4

We observe that *F*_(*ab*)_(*x*) is well-modeled by a quadratic function centered about the unconstrained free energy, *g*_(*ab*)_ = ΔΔ*G*_(*ab*)_^*^, and whose force constant is fit to 5 points *x* = ΔΔ*G*_(*ab*)_^*^ ± δ, where δ is 0, 1, or 2 kcal/mol. The graph objective and its solution for the ligand free energies are shown in eqs 5 and 6, respectively.

$$F\left(c\right)=\underset{\left(ab\right)}{\sum }\frac{k_{\left(ab\right)}}{2}{\left(c_{b}-c_{a}-g_{\left(ab\right)}\right)}^{2}$$ 5

$$c=M^{-1}\cdot X^{T}\cdot K\cdot g$$ 6

**g** is a *N*_edge_ × 1 array of unconstrained relative free energies, **K** is a *N*_edge_ × *N*_edge_ diagonal matrix of force constants, *K*_(*ab*),(*cd*)_ = δ_(*ab*),(*cd*)_ *k*_(*ab*)_, **X** is a *N*_edge_ × (*N*_lig_ – 1) matrix, *X*_(*ab*),*c*_ = δ_*bc*_ – δ_*ac*_, and **M** = **X** ^*T*^·**K**·**X**. One may have accurate reference (experimental) values for a subset of the edges. These can be incorporated as linear constraints, as described in the Supporting Information.

The edgembar program analyzes simulation data for a single edge. It computes the state free energies and pretabulates the effective edge objective function (eq 4). The input is a XML file which organizes the simulation data into the hierarchy of environments, stages, trials, and states. The data from a trial are a collection of files named: “efep_*tlam*_*elam*.dat”, where *tlam* is the sampled state, and *elam* is the state whose potential energies are tabulated within the file. The first column is the simulation time (ps), and the second column is a potential energy (kcal/mol). Further discussion and examples can be found in the Supporting Information and Quick Start Tutorial.^31^ The edgembar output is organized into a data structure and written to a python file that can be imported directly into other scripts for analysis. Execution of the python output causes its results to be summarized in a HTML-formatted “edge report”.

The edgembar-WriteGraphHtml.py script reads multiple edgembar outputs, calculates the ligand free energies (eq 6), and summarizes the results in a HTML-formatted “graph report”, which compares the isolated edge free energies to the ligand free energy differences. Tables of closed paths and their closure errors are included. The graph and edge reports display energies in kcal/mol; however, future releases of edgembar will allow one to choose the output energy units. Reanalysis of the ligand free energies is inexpensive when new data is introduced because only the new edges need to be recalculated; the cost of solving the ligand free energies from eq 6 is small.

There are several existing python-based MBAR implementations for calculating state free energies in a trial.^58–60^ edgembar is a C++ implementation that supports OpenMP parallelization but lacks GPU acceleration. The key feature of edgembar is its ability to simultaneously solve for all trials, stages, and environments while imposing constraints on the resulting ΔΔ*G* to precalculate edge objective functions for networkwide analysis.

## Alchemical λ Schedules

The fetkutils component supplies the fetkutils-tischedule.py script for preparing application-specific AFE λ-schedules^5^ to improve phase space overlap and the efficiency of Hamiltonian replica exchange (HRE). The MBAR method requires phase space overlap between states to produce reliable results.^9^ Furthermore, poor overlap between any pair of adjacent states produces an exchange bottleneck in HRE simulations that adversely effect round-trip statistics.^61,62^

For a given number of states, the goal is to choose the simulated λ values to achieve uniform exchange rates or phase space overlap along the λ coordinate. To do this, one simulates an alchemical transformation for a brief amount of time with a large number of alchemical states (for example, 21 states) to ensure good phase space overlap between adjacent states. One then chooses a schedule size for production, and the scheduling script analyzes the “burn-in” simulations to optimize the λ values to minimize the variance in a property along the alchemical dimension (either the predicted replica exchange probability ratios, phase space overlap, or Kullback–Leibler divergence). An extensive discussion of the underlying theory is found in ref 5 and the Supporting Information. In addition to choosing the schedule size and property, one can also place conditions on the optimized schedule, such as enforcing symmetry about λ = 0.5.

## Conclusions and Outlook

As free energy simulation methods advance to tackle increasingly complex problems, there is great need to develop robust, automated, efficient, and scalable analysis methods able to keep pace. These tools are critical to inform users of potential issues and provide data analytics needed to troubleshoot. FE-ToolKit was created to address these challenges and will continue to be developed and maintained to support emerging integrated free energy methods.

## Supplementary Material



## Data Availability

FE-ToolKit software, full documentation, and a quick start guide are distributed under the MIT License at https://gitlab.com/RutgersLBSR/fe-toolkit or as part of the AmberTools package available at https://ambermd.org/AmberTools.php.

## Notes

A detailed discussion of the theory, algorithms, error analysis, file formats, and examples (PDF)
