# Supplementary material for: FE-ToolKit: A Versatile Software Suite for Analysis of High-Dimensional Free Energy Surfaces and Alchemical Free Energy Networks
Source: J Chem Inf Model. 2025 May 21;65(11):5273–9. doi: 10.1021/acs.jcim.5c00554 (PMC12232920; doi:10.1021/acs.jcim.5c00554)
Supplement: Supplementary file 1 [file ci5c00554_si_001.pdf]

Supporting information for: FE-ToolKit: A versatile software suite for analysis of high-dimensional free energy surfaces and alchemical free energy networks

Timothy J. Giese, Ryan Snyder, Zeke Piskulich, German P. Barletta, Shi Zhang, Erika McCarthy, Şölen Ekesan, and Darrin M. York*

Laboratory for Biomolecular Simulation Research, Institute for Quantitative Biomedicine and Department of Chemistry and Chemical Biology, Rutgers University, Piscataway, NJ 08854, USA

E-mail: Darrin.York@rutgers.edu

Contents

1 Free energy surface analysis **S3**

1.1 Biasing potentials S3

1.2 High-dimensional potential of mean force profile construction S4

1.3 High-level free energy surface correction S5

1.4 Minimum free energy path determination S6

1.5 Visualization and error estimation S7

1.6 Generalized Bias Potentials S8

*To whom correspondence should be addressed

2 Alchemical free energy simulations **S9**

2.1 The EdgeMBAR method S9

2.2 Enforcing experimental (or reference) relative free energies S15

2.3 Edge properties tabulated during networkwide analysis S15

2.4 Error analysis S16

3 Tools for optimizing alchemical free energy λ-schedules **S17**

4 Automatic detection of unequilibrated data **S21**

5 File formats **S23**

5.1 `ndfes` metafile format S23

5.2 `ndfes` dumpave format S24

5.3 `ndfes` checkpoint file format S24

5.4 `ndfes-genbias` metafile format S26

5.5 `ndfes-genbias` dumpave format S27

5.6 `edgembar` XML input S28

5.7 `edgembar` raw data files S29

6 Examples **S29**

6.1 1-dimensional FES calculated from vFEP and MBAR S29

6.2 Analyzing aggregate sampling and updating errors to account for the variation between trials S31

6.3 2-dimensional FES calculated from MBAR S32

6.4 Calculation of an alchemical transformation free energy S33

6.5 Networkwide analysis of alchemical free energies S41

1 Free energy surface analysis

1.1 Biasing potentials

The functional form of the harmonic biasing potential adopted by the `ndfes` program is given by eq. 1, where d indexes the reaction coordinates, ξ_d is a reaction coordinate value, $\xi_{0,d}$ is an umbrella position, and k_d is a harmonic prefactor.

$$W(\boldsymbol{\xi}) = \sum_{d=1}^{N_{\text{dim}}} k_d(\xi_d - \xi_{0,d})^2 \tag{1}$$

Note that some analysis programs, such as Grossfield's `WHAM` program,[S1] use a traditional spring potential that includes a leading factor of $1/2$.

$$W(\boldsymbol{\xi}) = \sum_{d=1}^{N_{\text{dim}}} \frac{k_{\text{spr},d}}{2}(\xi_d - \xi_{0,d})^2 \tag{2}$$

The $k_{\text{spr},d}$ value is the spring constant (or force constant). The distinction of whether the leading factor of 1/2 is present often leads to confusion because many molecular dynamics programs, such as Amber, use the form shown in eq. 1. When writing the `ndfes` input files, the value of the k_d prefactor should be the same value as what was used in Amber's "disang" NMR restraint file format if the collective variable is a distance. In contrast, the input to Grossfield's `WHAM` program[S1] would be twice this value because eq. 2 includes a leading factor of 1/2. Special care must be taken by user's of the Amber software when restraining angles because the disang file lists the harmonic prefactor in units of kcal/mol/radian^2, whereas the collective variable timeseries is recorded in units of degrees. The `ndfes` program assumes angles are reported in degrees, and the prefactor is provided in units of kcal/mol/degree^2. In this case, the prefactor written to the `ndfes` input file should scale the value appearing within the disang file by a factor of $(\pi/180)^2 \approx 0.00030461741978670857$. Note that `FE-ToolKit` also provides a `ndfes-genbias` program that is similar to `ndfes`; however, it does not assume a functional form for the biasing potential. Instead, it manually reads the values of the biasing potentials from the input files.

1.2 High-dimensional potential of mean force profile construction

The variational free energy profile (vFEP) and multistate Bennett acceptance ratio (MBAR) methods are implemented in the `ndfes` program to produce multidimensional free energy surfaces from umbrella sampling. The vFEP method models the unbiased free energy surface of Hamiltonian h, $F_h(\boldsymbol{\xi};\mathbf{p})$ in a reduced set of reaction coordinates $\boldsymbol{\xi}$ using a global analytic function that contains parameters $\mathbf{p}$ which are chosen to maximize the log-likelihood function of the observed biased samples.

$$\mathbf{p}^* = \arg\max_{\mathbf{p}} \left\{ -\sum_{k=1}^{K_h} \left(\ln Z_{hk} + \frac{1}{N_{hk}} \sum_{n=1}^{N_{hk}} \beta F_{hk}(\boldsymbol{\xi}(\mathbf{r}_{hkn});\mathbf{p}) \right) \right\} \tag{3}$$

$\mathbf{r}_{hkn}$ is sample n drawn from the ensemble of potential h subjected to bias k, N_{hk} is the number of samples in the ensemble, K_h is the number of biasing potentials, $F_{hk}(\boldsymbol{\xi};\mathbf{p}) = F_h(\boldsymbol{\xi};\mathbf{p}) + W_{hk}(\boldsymbol{\xi})$ is the biased free energy surface, $W_{hk}(\boldsymbol{\xi})$ is the biasing potential, and Z_{hk} is a configuration integral.

$$Z_{hk} = \int \cdots \int \exp[-\beta F_{hk}(\boldsymbol{\xi};\mathbf{p})] d\xi_1 \cdots \xi_N \tag{4}$$

In contrast, the multistate Bennett acceptance ratio method constructs a free energy surface by reweighting the biased samples to approximate the unbiased probability distribution, $\rho_h(\xi)$.

$$F_h(\xi) = -\beta^{-1} \ln \rho_h(\xi) \tag{5}$$

The probability distribution is discretized on a multidimensional histogram.

$$\rho_h(\xi_m) = \sum_{k=1}^{K_h} \sum_{n=1}^{N_{hk}} \delta(\xi_m - \xi(\mathbf{r}_{hkn})) \omega_h(\mathbf{r}_{hkn}) \tag{6}$$

The subscript m indexes the histogram bins, such that ξ_m denotes a bin center.

$$\delta(\xi_m - \xi(\mathbf{r}_{hkn})) = \begin{cases} 1, & \text{if } -\Delta\xi/2 < \xi_m - \xi(\mathbf{r}_{hkn}) < \Delta\xi/2 \\ 0, & \text{otherwise} \end{cases} \tag{7}$$

The weight ω_h of sample $\mathbf{r}_{hkn}$ to the unbiased free energy is given by eq. 8, where U_h is an unbiased potential energy, U_{hk} is a biased potential energy, W_{hk} is the biasing potential, and the F_{hk} values are the free energies of each biased state, which are obtained by solving the MBAR/UWHAM equations.

$$\begin{aligned}\omega_h(\mathbf{r}_{hkn}) &= \frac{\exp[\beta F_h - \beta U_h(\mathbf{r}_{hkn})]}{\sum_{k'=1}^{K_h} N_{hk'} \exp[\beta F_{hk'} - \beta U_{hk'}(\mathbf{r}_{hkn})]} \\ &= \frac{\exp(\beta F_h)}{\sum_{k'=1}^{K_h} N_{hk'} \exp[\beta F_{hk'} - \beta W_{hk'}(\mathbf{r}_{hkn})]}\end{aligned} \tag{8}$$

The `ndfes` program input, referred to as a "metafile", lists several properties for each biased simulation, including: the simulation temperature, the unbiased Hamiltonian index used to perform the sampling, the harmonic force constant and location in each dimension, and a file name containing the biased sampling. The unbiased Hamiltonian index is only relevant when performing the weighted thermodynamic perturbation method[S2] (wTP) and generalized weighted thermodynamic perturbation method[S3] (gwTP) described in the next section. These two methods calculate the unbiased free energy surface of a target potential from the biased sampling produced from one-or-more reference potentials. The file containing the biased sampling, referred to as a "dumpave" file, contains $N_{\mathrm{dim}} + 1$ columns, where N_{dim} is the number of biasing potentials. The first column is the simulation time, and the remaining N_{dim} columns are the reaction coordinate values.

1.3 High-level free energy surface correction

The weighted thermodynamic perturbation method[S2] (wTP) and generalized weighted thermodynamic perturbation method[S3] (gwTP) apply the MBAR equations to estimate the unbiased free energy surface of a target potential energy function, $F_t(\xi)$ from the biased sampling performed with a reference potential (wTP) or multiple reference potentials (gwTP). Equation 9 expresses both wTP and gwTP, where N_{PE} is the number of reference potentials, and ω_t is the sample weight

of the unbiased target potential, t.

$$F_t(\xi_m) = -\beta^{-1}\ln \sum_{h=1}^{N_{\mathrm{PE}}} \sum_{k=1}^{K_h} \sum_{n=1}^{N_{hk}} \delta(\xi_m - \xi(\mathbf{r}_{hkn}))\omega_t(\mathbf{r}_{hkn}) \tag{9}$$

$$\omega_t(\mathbf{r}_{hkn}) = \frac{\exp[\beta F_t - \beta U_t(\mathbf{r}_{hkn})]}{\sum_{h'=1}^{N_{\mathrm{PE}}} \sum_{k'=1}^{N_{h'}} N_{h'k'} \exp[\beta F_{h'k'} - \beta U_{h'k'}(\mathbf{r}_{hkn})]} \tag{10}$$

The `ndfes` program requires the unbiased potential energy of each reference and target potential for every sample to evaluate the wTP or gwTP methods. These energies appear as extra columns in the dumpave file. Furthermore, one must know which potential was used to produce the sampling; this is the value of the "Hamiltonian index" appearing in the metafile. This index indicates which of the extra columns of unbiased potential energies is the reference potential that produced the samples.

1.4 Minimum free energy path determination

The surface-accelerated string method (SASM)[S4] differs from most other chain-of-states methods by decoupling the representation of the path from the umbrella sampling used to improve the free energy estimate. The aggregate sampling from the current and previous iterations is used to calculate a free energy surface in the reduced dimensional space of reaction coordinates. The best estimate of the minimum free energy path is optimized on that fixed surface. Additional sampling is then chosen to either improve the sampling near the path or by extending the range of the free energy surface in the direction that the path has moved from the previous best estimate. This strategy has been shown to accelerate the convergence of the path when the initial guess is far from the optimized path, and the method converges the free energy surface by incorporating the sampling from the current and previous iterations.

The `ndfes` package includes 3 programs for performing the SASM:

- `ndfes-path-analyzesims.py` This extracts the samples from current string iteration and prepares a ndfes metafile used to calculate a free energy surface from the current and previous

iterations.

- `ndfes` This is the main driver program that analyzes the umbrella sampling, solves the MBAR equations, and saves the free energy surface to a checkpoint file.

- `ndfes-path` This program reads a free energy surface checkpoint file, optimizes a minimum free energy path, and writes a new set of simulation input files for the next string iteration.

1.5 Visualization and error estimation

The `ndfes` package offers several metrics to estimate uncertainties. The `ndfes` program will perform cyclic block bootstrap analysis of the samples to report a standard error for each bin. The block size is chosen from the biasing potential's autocorrelation time, and the number of bootstrap samples is chosen by setting the `--nboot` command–line option when executing `ndfes`. Each bootstrap calculation resamples each simulation with replacement. The FES is calculated from the resampled simulations. Many bootstrap calculations are performed to produce a distribution of free energy values for each histogram bin. The standard error of a FES bin value is the standard deviation of the bootstrap distribution. In some instances, the user may wish to generate a series of free energy surfaces from independent sets of simulations. The `ndfes-AvgFESs.py` program will average multiple free energy surfaces and adjust the uncertainties to account for the variance between the trials. The surface produced by each trial, $F_t^{(i)}(\xi_m)$, is defined to within a constant C_i which is chosen to minimize the weighted square differences with respect to the first trial.

$$\frac{\partial}{\partial C_i} \sum_m w_m^{(i)} \left([F_t^{(i)}(\xi_m) + C_i] - F_t^{(1)}(\xi_m) \right)^2 = 0 \tag{11}$$

The first trial's constant is zero $C_1 = 0$ and the weights are chosen to be inversely proportional to the individual trial's bootstrap variances, $\delta F_t^{(i)}(\xi_m)^2$.

$$w_m^{(i)} = \frac{\left(\delta F_t^{(i)}(\xi_m)^2 + \delta F_t^{(1)}(\xi_m)^2 \right)^{-1}}{\sum_n \left(\delta F_t^{(i)}(\xi_n)^2 + \delta F_t^{(1)}(\xi_n)^2 \right)^{-1}} \tag{12}$$

The trial-averaged surface and uncertainty are given by eqs. 13 and 14.

$$\bar{F}_t(\xi_m) = \frac{1}{N_{\text{trial}}} \sum_{i=1}^{N_{\text{trial}}} F_t^{(i)}(\xi_m) + C_i \tag{13}$$

$$\delta\bar{F}_t(\xi_m) = \left(\frac{1}{N_{\text{trial}}} \sum_{i=1}^{N_{\text{trial}}} \frac{\left[F_t^{(i)}(\xi_m) + C_i - \bar{F}_t(\xi_m)\right]^2}{N_{\text{trial}} - 1} + \frac{\left[\delta F_t^{(i)}(\xi_m)\right]^2}{N_{\text{trial}}} \right)^{1/2} \tag{14}$$

The reliability of the wTP and gwTP reweighted samples are judged by reporting a "reweighting entropy" for each bin (eq. 15).

$$\mathcal{S}_t(\xi_m) = -\frac{\sum_{h=1}^{N_{\text{PE}}} \sum_{k=1}^{K_h} \sum_{n=1}^{N_{hk}} \delta(\xi_m - \xi(\mathbf{r}_{hkn})) \frac{\omega_t(\mathbf{r}_{hkn})}{s_{tm}} \ln \frac{\omega_t(\mathbf{r}_{hkn})}{s_{tm}}}{\ln \sum_{h=1}^{N_{\text{PE}}} \sum_{k=1}^{K_h} \sum_{n=1}^{N_{hk}} \delta(\xi_m - \xi(\mathbf{r}_{hkn}))} \tag{15}$$

$$s_{tm} = \sum_{h=1}^{N_{\text{PE}}} \sum_{k=1}^{K_h} \sum_{n=1}^{N_{hk}} \delta(\xi_m - \xi(\mathbf{r}_{hkn}))\omega_t(\mathbf{r}_{hkn}) \tag{16}$$

The reweighting entropy is a number between 0 and 1 indicating whether the sample weights are uniform (a reweighting entropy close to 1) or dominated by only a few samples (an entropy close 0).

The bin centers, free energy values, standard errors, bin sample sizes, and reweighting entropies are stored in an Extensible Markup Language (XML) checkpoint file. The data can be queried and visualized using the `ndfes` python companion library. The raw data can be printed using the `ndfes-PrintFES.py` script, and the `FE-ToolKit` software includes examples that plot 2- and 3-dimensional free energy surfaces.

1.6 Generalized Bias Potentials

The `ndfes` program assumes that the bias is a harmonic function, which is often adequate for most free energy applications. `FE-ToolKit` provides a separate program called `ndfes-genbias` that can perform MBAR, wTP, and gwTP analysis using general biasing functions. Rather than specifying the harmonic potential prefactors and positions in the metafile for each state, one in-

stead manually includes extra columns of bias potential energies within the dumpave files. The `ndfes-genbias` metafile includes an extra 0-based integer called the "bias index" that denotes which of the extra columns of bias potentials corresponds to the bias used during sampling. As mentioned, the metafile and dumpave file formats are different when using the `ndfes-genbias` program. The formats are specified in section 5.4.

The `ndfes-genbias` program cannot evaluate the vFEP method because the vFEP method performs numerical integration, which requires knowledge of the bias potential values at the quadrature roots. These values are not contained with the dumpave files. In contrast, the `ndfes` program can directly evaluate the bias at the quadrature roots from the analytic form. We do not recommend using the `ndfes-genbias` program unless it is absolutely necessary because it will require writing very large input files and a large memory workstation when trying to use it in applications that involve a million or more samples aggregated from a thousand or more states. Furthermore, the sampling generated from multiple trials and/or reference potentials cannot be easily aggregated by concatenating their metafiles. When metafiles contain different biasing potentials, the "bias indexes" in each are invalid when concatenating them. Similarly, the dumpave files would need to be completely rewritten to account for the additional columns of biasing potentials introduced by the other metafile.

2 Alchemical free energy simulations

2.1 The EdgeMBAR method

Alchemical free energy simulations can be visualized as a graph (network) whose nodes and edges represent ligands and AFE transformations, respectively. The free energy of an edge connecting ligands a and b is a relative free energy because it is the difference between the transformation free energies in two environments: the target and reference environments.

$$\Delta\Delta G_{(ab)} = \Delta G_{(ab),\text{target}} - \Delta G_{(ab),\text{ref}} \tag{17}$$

When calculating relative solvation free energies, the target and reference states are aqueous and gas phase, respectively. When calculating relative binding free energies, the target and reference states are the protein–bound and unbound (aqueous) phase. Calculation of absolute solvation and binding free energies similarly involves 2 environments; however, the graph consists of only 2 ligands: the real ligand and a “dummy state” ligand that does not interact with its environment.

The difference in ligand free energies within environment e, $\Delta G_{(ab)e}$, is calculated from 1-or-more stages that alchemically transform ligand a to b. The transformation is typically performed using either a concerted (1-stage) or stepwise (3-stage) protocol. The stepwise protocol (sometimes called a multistep or split procedure[S5]) has traditionally been used to avoid “endpoint catastrophes”[S6–S9] by decomposing the particle creation and annihilation processes into electrostatic and nonelectrostatic processes. Alternatively, softcore potentials were designed to reduce instabilities encountered in 1-stage creation/annihilation processes.[S9] The softcore potentials, like the smoothstep softcore method,[S10] continue to be developed to improve the stability of alchemical free energy simulations. Let $\Delta G_{(ab)es}$ and N_{stage} denote the free energy calculated from stage s and the number of stages. The free energy difference in environment e, $\Delta G_{(ab)e}$, is the sum of the stage free energies, $\Delta G_{(ab)es}$.

$$\Delta G_{(ab)e} = \sum_{s=1}^{N_{\text{stage}}} \Delta G_{(ab)es} \tag{18}$$

The alchemical simulations are often repeated by varying the initial conditions or thermostat random seed value. The stage free energy is an average of the independent trials, where t indexes the trial and $N_{\text{trial},(ab)es}$ is the number of trials.

$$\Delta G_{(ab)es} = \frac{1}{N_{\text{trial},(ab)es}} \sum_{t=1}^{N_{\text{trial},(ab)es}} \Delta G_{(ab)est} \tag{19}$$

The stage free energy of a trial is the difference between the final $G_{(ab)est,\lambda=1}$ and starting $G_{(ab)est,\lambda=0}$ states, where λ is the alchemical parameter.

$$\Delta G_{(ab)est} = G_{(ab)est,\lambda=1} - G_{(ab)est,\lambda=0} \tag{20}$$

The value of λ is required to define the potential energy function, $U_{(ab)es}(\mathbf{r};\lambda)$, where $\mathbf{r}$ is the $3N$ array of Cartesian coordinates. Equilibrium sampling of $U_{(ab)es}(\mathbf{r};\lambda)$ at a discrete series of $N_{\text{state},(ab)est}$ λ-values spanning $0 \leq \lambda \leq 1$ produces a corresponding series of ensembles. Let $\mathbf{r}_{(ab)estjk}$ denote the coordinate array of sample k in ensemble j generated from simulation trial t. The remaining subscripts identify the ensemble as an alchemical state in stage s of the transformation between between physical states $a \rightarrow b$ performed in environment e. The ensembles produced from trial t can be analyzed with the MBAR method to calculate the free energies of the $N_{\text{state},est}$ alchemical states. The MBAR solution is a $N_{\text{state},est}\times 1$ array of values, $\mathbf{G}_{(ab)est}$, found by minimizing the convex objective function, $F_{(ab)est}$.

$$F_{(ab)est}(\mathbf{G}_{(ab)est}) = \frac{1}{N_{\text{s},(ab)est}} \sum_{j=1}^{N_{\text{s},(ab)est}} \sum_{k=1}^{N_{\text{s},(ab)estj}} \ln\left(\sum_{l=1}^{N_{\text{state},(ab)est}} \exp\left[-\beta U_{(ab)es}(\mathbf{r}_{(ab)estjk};\lambda_l) - b_{(ab)estl}\right]\right) + \sum_{i=1}^{N_{\text{state},(ab)est}} \frac{N_{\text{s},(ab)esti}}{N_{\text{s},(ab)est}} b_{(ab)esti} \tag{21}$$

$N_{\text{s},(ab)esti}$ is the number of samples drawn from the state λ_i, $N_{\text{s},(ab)est}$ is the aggregate number of samples from the $N_{\text{state},(ab)est}$ states (eq. 22), and $b_{(ab)esti}$ is given by eq. 23.

$$N_{\text{s},(ab)est} = \sum_{i=1}^{N_{\text{state},(ab)est}} N_{\text{s},(ab)esti} \tag{22}$$

$$b_{(ab)esti} = -\ln\frac{N_{\text{s},(ab)esti}}{N_{\text{s},(ab)est}} - \beta G_{(ab)esti} \tag{23}$$

An edge consists of $N_{\text{state},(ab)}$ total states, whose free energies can be organized into a $N_{\text{state},(ab)}\times 1$ array, $\mathbf{G}_{(ab)}$.

$$N_{\text{state},(ab)} = \sum_{e} \sum_{s=1}^{N_{\text{stage}}} \sum_{t=1}^{\text{trial},(ab)es} N_{\text{state},(ab)est} \tag{24}$$

The $\mathbf{G}_{(ab)}$ values can be determined from independent MBAR objective function minimizations of each trial, as previously described. Alternatively, one can define a global objective function for the

entire edge, $F_{(ab)}(\mathbf{G}_{(ab)})$. The edge objective function is the sum of trial objective functions.

$$F_{(ab)}(\mathbf{G}_{(ab)}) = \sum_{e} \sum_{s=1}^{N_{\text{stage}}} \frac{\sum_{t=1}^{N_{\text{trial},(ab)es}} F_{(ab)est}(\mathbf{G}_{(ab)est})}{N_{\text{trial},(ab)es}} \tag{25}$$

The $N^{-1}_{\text{trial},(ab)es}$ normalization factor is chosen to prevent $F_{(ab)}$ from scaling with the number of independent trials. The minimization of $F_{(ab)}$ is equivalent to independent minimizations of the trial objective functions if their solutions are are not subjected to constraints. The state free energies which minimize $F_{(ab)}$ shall be denoted with an asterisk.

$$\mathbf{G}^{*}_{(ab)} = \underset{\mathbf{G}_{(ab)}}{\arg\min}\ F_{(ab)}(\mathbf{G}_{(ab)}) \tag{26}$$

The edge's relative energy is computed from the optimized state free energies $\Delta\Delta G^{*}_{(ab)} \equiv \Delta\Delta G_{(ab)}(\mathbf{G}^{*}_{(ab)})$ using eqs. 17-20.

The MBARnet network analysis presented in Ref. S11 proceeds by constructing a graph objective function consisting of all edges.

$$F_{\text{MBARnet}}(\mathbf{G}) = \sum_{(ab)} F_{(ab)}(\mathbf{G}_{(ab)}) \tag{27}$$

The graph objective is minimized while imposing constraints on the solution to enforce cycle closure conditions. The MBARnet analysis has several disadvantages. It requires a large amount of computer memory to store all the simulation data for every edge. The computational cost of minimizing $F(\mathbf{G})$ becomes expensive as the size of the network increases. Furthermore, the entire network needs to be reoptimized if an edge is added or deleted from the network. Finally, the MBARnet method enforced cycle closure conditions only for minimum-length cycles (that is, cycles that cannot be traversed from the union of two smaller cycles). Enforcing these conditions does not strictly enforce the closure of larger cycles.

The EdgeMBAR method rectifies these shortcomings. The computational savings is achieved by analyzing each edge in isolation and modeling the behavior of the objective function in the

vicinity of the unconstrained relative free energy. In other words, one replaces the edge objective functions in eq. 27 with effective objective functions, $\tilde{F}_{(ab)}$, that explicitly depend on the edge free energy. (The argument of $F_{(ab)}$ is an array of state free energies, whereas the argument of $\tilde{F}_{(ab)}$ is a scalar edge free energy value.)

$$F(\mathbf{G}) = \sum_{(ab)} \tilde{F}_{(ab)}(\Delta\Delta G_{(ab)}(\mathbf{G}_{(ab)})) \tag{28}$$

The effective objective function is a tabulation of $F_{(ab)}$ values from a series of minimizations subjected to a constraint: $\Delta\Delta G_{(ab)}(\mathbf{G}_{(ab)}) = x$, where x is a target free energy value.

$$\begin{aligned} \tilde{F}_{(ab)}(x) =& \min_{\mathbf{G}_{(ab)}} F_{(ab)}(\mathbf{G}_{(ab)}) \\ & \text{subject to: } \Delta\Delta G_{(ab)}(\mathbf{G}_{(ab)}) = x \end{aligned} \tag{29}$$

We have observed that $\tilde{F}_{(ab)}(x)$ is well-modeled by a quadratic function. The location of the quadratic's minimum, $g_{(ab)}$, is the unconstrained relative free energy $g_{(ab)} = \Delta\Delta G^*_{(ab)}$. The quadratic's force constant is numerically fit to discrete values of $\tilde{F}_{(ab)}(x)$. In the present work, we tabulate $\tilde{F}_{(ab)}(x)$ at 5 points: $x = \Delta\Delta G^*_{(ab)} \pm \delta$, where δ is 0, 1, or 2 kcal/mol.

$$\tilde{F}_{(ab)}(x) \approx F_{(ab)}(\mathbf{G}^*_{(ab)}) + \frac{k_{(ab)}}{2}(x - g_{(ab)})^2 \tag{30}$$

The model form of the effective edge objective functions are used to define a networkwide objective function, $F_{\text{graph}}(\mathbf{c})$. The argument, $\mathbf{c}$ is a $(N_{\text{lig}} - 1) \times 1$ array of ligand free energies relative to a reference ligand. The free energy of the reference ligand, ΔG_0, is chosen to define the arbitrary zero of energy.

$$c_a = \Delta G_a - \Delta G_0 \tag{31}$$

$$
\begin{aligned}
F_{\mathrm{graph}}(\mathbf{c}) &= \sum_{(ab)}^{N_{\mathrm{edge}}} \tilde{F}_{(ab)}(c_b - c_a) \\
&= \sum_{(ab)}^{N_{\mathrm{edge}}} \frac{k_{(ab)}}{2} \left(c_b - c_a - g_{(ab)}\right)^2
\end{aligned}
\tag{32}
$$

By expressing F_{graph} in terms of the effective edge objective functions, the optimization reduces to choosing an appropriate set of ligand free energies because the effective edge objective functions depend solely on the ligand free energy differences: $\Delta\Delta G_{(ab)} = c_b - c_a$.

The graph objective function can be written in linear algebraic form, where $\mathbf{g}$ is a $N_{\mathrm{edge}} \times 1$ array of unconstrained relative free energies, $\mathbf{K}$ is a $N_{\mathrm{edge}} \times N_{\mathrm{edge}}$ diagonal matrix of edge objective function force constants, and $\mathbf{X}$ is a $N_{\mathrm{edge}} \times (N_{\mathrm{lig}} - 1)$ matrix that expresses the edge free energies from the relative ligand free energies.

$$
\begin{aligned}
F_{\mathrm{graph}}(\mathbf{c}) = &- \mathbf{c}^T \cdot \mathbf{X}^T \cdot \mathbf{K} \cdot \mathbf{g} \\
&+ \frac{1}{2}\mathbf{c}^T \cdot \mathbf{X}^T \cdot \mathbf{K} \cdot \mathbf{X} \cdot \mathbf{c} \\
&+ \frac{1}{2}\mathbf{g}^T \cdot \mathbf{K} \cdot \mathbf{g}
\end{aligned}
\tag{33}
$$

$$
K_{(ab),(cd)} = \delta_{(ab),(cd)} k_{(ab)} \tag{34}
$$

$$
X_{(ab),c} = \delta_{bc} - \delta_{ac} \tag{35}
$$

The relative ligand free energies are calculated from a linear algebraic solution.

$$
\mathbf{c} = \mathbf{M}^{-1} \cdot \mathbf{X}^T \cdot \mathbf{K} \cdot \mathbf{g} \tag{36}
$$

$$
\mathbf{M} = \mathbf{X}^T \cdot \mathbf{K} \cdot \mathbf{X} \tag{37}
$$

The ligand free energy uncertainties are estimated from bootstrap analysis of eq. 36 drawing samples of the **g** values from a normal distribution whose standard deviation is the standard error of the unconstrained edge free energies. The uncertainty in the edge free energies are propagated from the relative ligand uncertainties.

2.2 Enforcing experimental (or reference) relative free energies

In some instances, one may have accurate reference values (or experimental values) for a subset of the edges in the graph. One can incorporate the known free energy differences into the solution of the ligand free energies by using Lagrange's method of undetermined multipliers. In this case, the **c** values are chosen to minimize the following equation.

$$\frac{\partial}{\partial c_i}\left\{F_{\text{graph}}(\mathbf{c}) - \left(\mathbf{c}^T \cdot \mathbf{D}^T - \mathbf{v}^T\right)\cdot \mathbf{l}\right\} = 0 \tag{38}$$

$\mathbf{l}$ is a $N_{\text{ref}} \times 1$ array of Lagrange multipliers. $\mathbf{v}$ is a $N_{\text{ref}} \times 1$ array of reference edge free energy values. $\mathbf{D}$ is a $N_{\text{ref}} \times (N_{\text{lig}} - 1)$ matrix that expresses the target edge free energy in terms of the ligand free energies.

$$D_{(ab),c} = \delta_{bc} - \delta_{ac} \tag{39}$$

The ligand free energies and Lagrange multipliers obtained from solution of eq. 38 are given by eqs. 40 and 41, respectively.

$$\mathbf{c} = \mathbf{M}^{-1}\cdot\left(\mathbf{X}^T\cdot\mathbf{K}\cdot\mathbf{g} - \mathbf{D}^T\cdot\mathbf{l}\right) \tag{40}$$

$$\mathbf{l} = \left(\mathbf{D}\cdot\mathbf{M}^{-1}\cdot\mathbf{D}^T\right)^{-1}\cdot\left(\mathbf{D}\cdot\mathbf{M}^{-1}\cdot\mathbf{X}^T\cdot\mathbf{K}\cdot\mathbf{g} - \mathbf{v}\right) \tag{41}$$

2.3 Edge properties tabulated during networkwide analysis

When `edgembar` performs the networkwide analysis, it will summarize the edge free energies and other edge properties which may be useful in identifying edges that may be in error. These

properties are “shift”, “force constant”, “Lagrange multiplier index” (LMI), and “average cycle closure error” (ACC).

The shift is the absolute difference between the edge free energy calculated in isolation (eq. 26) and from networkwide analysis (eq. 32), $\Delta_{ab} = |\Delta\Delta G(\mathbf{G}^{*}) - (c_b - c_a)|$. If the isolated edge calculations were consistent across the network, such that the free energy along all closed paths is zero, then the shifts would also be zero.

The force constant is the quadratic prefactor within the effective edge objective function (eq. 30).

The LMI is the value of the Lagrange multiplier (eq. 41) needed to minimize $F_{\text{graph}}(\mathbf{c})$ while being subjected to the constraint: $c_b - c_a = \Delta\Delta G_{ab}(\mathbf{G}^{*})$. In other words, the solution for the ligand free energies is constrained to reproduce an *isolated* edge free energy. The calculation of the LMI values requires a separate minimization of $F_{\text{graph}}(\mathbf{c})$ for each edge.

The ACC of an edge is the average cycle closure error of all unique closed paths that traverse the edge. The cycle closure error is the absolute value of the free energy along a closed path, as calculated from the isolated edge free energies.

2.4 Error analysis

The `edgembar` program contains a `--nboot` command–line option to control the number of bootstrap calculations used to estimate the uncertainty in the state free energies. The bootstrap procedure resamples each simulation with replacement and solves the MBAR equations to obtain a new set of state free energies. The bootstrap calculation is repeated many times to produce a distribution of free energy values for each state. The standard error of each state free energy is the standard deviation of the bootstrap distribution, $G_{(ab)est,\lambda} \pm \delta G_{(ab)est,\lambda}$. The zero of free energy is arbitrary; therefore, we impose the condition $G_{(ab)est,\lambda=0} = 0$ for every trial, stage, and environment – including the bootstrap calculations. By doing so, the uncertainty of the $\lambda = 0$ state is always zero, $\delta G_{(ab)est,\lambda=0} \equiv 0$, and the uncertainty in the stage free energy of a trial (eq. 20) is the same as

the uncertainty of the $\lambda = 1$ state.

$$\delta\Delta G_{(ab)est} = \delta G_{(ab)est,\lambda=1} \tag{42}$$

The uncertainty in the trial–averaged stage free energy includes contributions from the variance between the trials and the bootstrap uncertainties caused by fluctuations within a trial.

$$\delta\Delta G_{(ab)es} = \left(\frac{1}{N_{\text{trial},(ab)es}} \sum_{t=1}^{N_{\text{trial},(ab)es}} \frac{\left[\Delta G_{(ab)est} - \Delta G_{(ab)es}\right]^2}{N_{\text{trial},(ab)es} - 1} + \frac{\left[\delta\Delta G_{(ab)est}\right]^2}{N_{\text{trial},(ab)es}} \right)^{1/2} \tag{43}$$

The uncertainty in the transformation free energy within environment e (eq. 44) and the edge (eq. 45) are propagated from the trial-averaged uncertainties.

$$\delta\Delta G_{(ab)e} = \left(\sum_{s=1}^{N_{\text{stage},(ab)e}} \left[\delta\Delta G_{(ab)es}\right]^2 \right)^{1/2} \tag{44}$$

$$\delta\Delta G_{(ab)} = \left(\sum_{e} \left[\delta\Delta G_{(ab)e}\right]^2 \right)^{1/2} \tag{45}$$

3 Tools for optimizing alchemical free energy λ-schedules

The convergence of alchemical free energy simulations is aided by the use of Hamiltonian replica exchange molecular dynamics (HREMD), which enhances the sampling by allowing traversal across potential energy barriers separating thermally relevant minima. The exchange efficiency of HREMD, as measured by the number of replica "round trips", is largely determined by the similarity between the potential energies of neighboring λ-states, where a round trip is the traversal of a walker from the $\lambda = 0$ to $\lambda = 1$ state and back to the $\lambda = 0$ state. Ideally each consecutive gap between λ-states would have very similar exchange acceptance frequencies, because the number of round trips is limited by the gap with the smallest acceptance frequency. One approach for increasing the acceptance rates would be to simply use more λ-states; however, this approach can

lead to fewer observed round trips because there are now more alchemical states to traverse. A more cost-effective approach would be to modify the schedule of λ-states by increasing the gap between adjacent states whose acceptance rate was larger than the average while similarly reducing the gap between neighbors whose acceptance rate was lower than the average. To apply this strategy, we perform brief "burn-in" simulations with a uniformly spaced schedule to gather preliminary statistics. We analyze the simulations to predict the acceptance ratio (AR, eq. 46) between each pair of states. A model function is then parametrized to predict the acceptance ratio between arbitrary pairs of states, and this function is used in a nonlinear optimization to choose a schedule for production sampling. The optimized schedule is the one that predicts a uniform acceptance ratio between each pair of neighboring states.

The acceptance ratio is calculated from the Metropolis criterion at constant temperature.

$$\begin{aligned} p_{ijn} =& \min(1, e^{-\beta\left[\left\{U(\mathbf{r}_{jn};\lambda_i)+U(\mathbf{r}_{in};\lambda_j)\right\}-\left\{U(\mathbf{r}_{in};\lambda_i)+U(\mathbf{r}_{jn};\lambda_j)\right\}\right]}) \\ =& \min\left(1, \frac{e^{-\beta\Delta U_{ij}(\mathbf{r}_{jn})}}{e^{-\beta\Delta U_{ij}(\mathbf{r}_{in})}}\right) \end{aligned} \tag{46}$$

The "AR index" is defined to be the average exchange probability.

$$O_{ij} = \frac{1}{\min(M_i, M_j)} \sum_{n=1}^{\min(M_i,M_j)} p_{ijn} \tag{47}$$

The AR index values obtained from the burn-in simulations produce a symmetric matrix O_{ij}. These values are used to concoct a continuous function $O(\lambda_i, \lambda_j)$ for predicting AR index values between states that have not been explicitly simulated. The continuous function is parametrized to satisfy $O(\lambda_i, \lambda_j) = O_{ij}$ and $O(\lambda, \lambda) = 1 \; \forall \; \lambda$. The second condition is enforced by introducing a coordinate transformation (eqs. 48-49) and model functional form (eq. 50).

$$u(\lambda, \lambda') = (\lambda - \lambda')/\sqrt{2} \tag{48}$$

$$v(\lambda, \lambda') = (\lambda + \lambda')/\sqrt{2} \tag{49}$$

$$O(\lambda, \lambda') = e^{-z(u(\lambda,\lambda'),v(\lambda,\lambda'))|u(\lambda,\lambda')|} \tag{50}$$

The exponent $z(u, v)$ appearing in eq. 50 is represented by a multiquadric radial basis function.

$$z(u, v) = \sum_{ij} w_{ij}\varphi(\sqrt{(u - u_{ij})^2 + (v - v_{ij})^2}) \tag{51}$$

The radial basis function $\varphi(r) = \sqrt{1 + (\epsilon r)^2}$, depends on a "shape parameter", $\epsilon = 100$. The u_{ij} and v_{ij} values correspond to the locations of the burn-in simulations.

$$u_{ij} = u(\lambda_i, \lambda_j) \tag{52}$$

$$v_{ij} = v(\lambda_i, \lambda_j) \tag{53}$$

The weights, w_{kl} are chosen to reproduce the AR indexes by solving a system of linear equations.

$$\sum_{kl} A_{(ij),(kl)} w_{kl} = z_{ij} \tag{54}$$

The z_{ij} values are set of exponents that reproduce the AR indexes from eq. 50.

$$z_{ij} = -\ln(O_{ij})/|u_{ij}| \tag{55}$$

A is a positive definite matrix of radial basis functions.

$$A_{(ij),(kl)} = \varphi(\sqrt{(u_{ij} - u_{kl})^2 + (v_{ij} - v_{kl})^2}) \tag{56}$$

The parametrized model for the AR indexes is used to optimize a schedule of λ values. The

objective function (eq. 57) is the variance of AR indexes between adjacent states.

$$\chi^2(\boldsymbol{\lambda}) = \frac{1}{N_\lambda - 1} \sum_{i=1}^{N_\lambda - 1} [O(\lambda_i, \lambda_{i+1}) - \langle O(\boldsymbol{\lambda}) \rangle]^2 \tag{57}$$

The objective function is a minimum when every neighboring AR index is the same as the average AR index.

$$\langle O(\boldsymbol{\lambda}) \rangle = \frac{1}{N_\lambda - 1} \sum_{i=1}^{N_\lambda - 1} O(\lambda_i, \lambda_{i+1}) \tag{58}$$

The analysis of the burn-in simulations and optimization of a λ-schedule is performed with `fetkutils-tischedule.py`. The recommended analysis uses the replica exchange acceptance ratios, as we've just described; however, other options exist, such as the analysis of the "phase space overlap" and "Kullback-Leibler divergence"[S12] discussed in ref. S13. The optimizations are performed with the low-storage Broyden-Fletcher-Goldfarb-Shanno algorithm[S14] implemented in the SciPy software.[S15]

The default optimization procedure is to fix the $\lambda = 0$ and $\lambda = 1$ states and optimize the remaining $N - 2$ values; however, additional constraints on the solution can be applied. The `--sym` command-line option will force the schedule to be symmetric about $\lambda = 0.5$. Furthermore, one can reduce the number of free parameters by using a variable smoothstep softcore (SSC) schedule.[S10] Reference S10 introduced a family of SSC polynomials, $S_n(\lambda)$, that obey $S_n(0) = 0$ and $S_n(1) = 1$, where the polynomial order is $2n + 1$. High order SSC polynomials enforce successively higher derivative conditions at the $\lambda = 0$ and $\lambda = 1$ endpoints. Specific examples include the S_1 and S_2 polynomials.

$$S_1(\lambda) = -2\lambda^3 + 3\lambda^2 \tag{59}$$

$$S_2(\lambda) = 6\lambda^5 - 15\lambda^4 + 10\lambda^3 \tag{60}$$

A "SSC schedule" is the series of λ values $(\lambda_0, \lambda_1, \cdots, \lambda_{N-2}, \lambda_{N-1})$ that uniformly discretize the $S_n(\lambda)$ polynomial inverse function. In other words, the set of λ_i values are determined by finding the root of the equation $S_n(\lambda_i) = i/(N-1)$, where N is the size of the schedule. One can generalize

the SSC polynomial by introducing a parameter α that takes a value $1 \leq \alpha \leq 2$.

$$S(\lambda;\alpha) = (2-\alpha)S_1(\lambda) + (\alpha-1)S_2(\lambda) \tag{61}$$

The α parameter continuously varies the polynomial between $S(\lambda;\alpha = 1) = S_1(\lambda)$ and $S(\lambda;\alpha = 2) = S_2(\lambda)$. The `fetkutils-tischedule.py` program can optimize a symmetric SSC schedule by varying a single parameter (α). The optimization can also be performed using a generalized 2–parameter SSC function to yield an asymmetric schedule.

$$\begin{aligned} S(\lambda;\alpha_0,\alpha_1) =& (1-\lambda)S(\lambda;\alpha_0) + \lambda S(\lambda;\alpha_1) \\ =& \left[(1-\lambda)(2-\alpha_0) + \lambda(2-\alpha_1)\right] S_1(\lambda) \\ &+ \left[(1-\lambda)(\alpha_0-1) + \lambda(\alpha_1-1)\right] S_2(\lambda) \end{aligned} \tag{62}$$

By optimizing a symmetric or asymmetric SSC schedule with a reduced number of parameters, the resulting schedule is less sensitive to the statistical noise encountered from the limited sampling obtained from the burn-in simulations.

4 Automatic detection of unequilibrated data

Umbrella sampling and alchemical free energy simulations involve analyzing many simulations. It is advantageous, therefore, to have a mechanism to automatically determine if the beginning of the simulation should be discarded due to the presence of unequilibrated sampling. To analyze an umbrella sampling simulation, we examine the bias energy as a function of time. In contrast, the alchemical free energy simulations are examined twice: once using the potential energy difference between adjacent alchemical states in the forward (eq. 63) direction and the potential energy difference in the reverse (eq. 64) direction. Two sets of analysis may detect different amounts of

simulation to exclude as equilibration, in which case we discard the larger percentage.

$$\Delta U_{\text{fwd},i}(t) = U(\mathbf{r}(t); \lambda_{i+1}) - U(\mathbf{r}(t); \lambda_i) \tag{63}$$

$$\Delta U_{\text{rev},i}(t) = U(\mathbf{r}(t); \lambda_{i-1}) - U(\mathbf{r}(t); \lambda_i) \tag{64}$$

We proceed by describing the algorithm for a generic time series of potential energies.

To begin, let $f_{\text{eq}} = 0$ be the fraction of data to excluded from the start of the time–series. After excluding the fraction f_{eq} as equilibration, the remaining samples are the proposed "production region". The goal is to determine whether the samples in the production region correspond to converged sampling; if not, then f_{eq} should be increased by an additional 5%. To test if the production region corresponds to converged sampling, we pose 3 tests. If any 2 of the 3 tests suggest the proposed production region contains ill-converged samples, then the f_{eq} is increased. The first test splits the proposed production region into 2 halves, and we perform Welch's t-test to query if the two halves produce statistically different means. If the t-test suggests the means are different, then the production region likely contains ill-converged sampling. The second test similarly splits the proposed production region into 2 halves, and if the two means differ by less than d_{tol} (a user–defined tolerance), then the two means are understood to effectively be the same value – even if the t-test suggests otherwise. The third test performs a linear regression through the data to detect a drift. A drift is detected via a two-tailed Wald Chi–Squared Test, which determines if there is evidence to suggest the slope is nonzero. The critical p–values used in the Welch and Wald tests and the d_{tol} tolerance can be chosen by the user. The default values are $p_{\text{tol}} = 0.05$ and $d_{\text{tol}} = 0.1$ kcal/mol.

5 File formats

5.1 `ndfes` metafile format

The `ndfes` program directly reads a "metafile". Each line of the metafile characterizes a biased state and provides a filename that contains the raw samples.

- Column 1: *integer*. This is a zero-based integer, known as the "Hamiltonian index". The value of the integer should be 0 unless one is calculating the wTP or gwTP methods. In these cases, the dumpave file contains 2-or-more extra columns of unbiased potential energies, and the Hamiltonian index indicates which extra column corresponds to the unbiased potential energy of the sampled state.

- Column 2: *float*. The simulation temperature in Kelvin. The original intention of including the temperature within the metafile was to allow for the possibility of simulating biased states at different temperatures and then analyzing the free energy surface at another temperature. This functionality has not been extensively tested, and initial tests have suggested that the free energy surfaces suffer from significant numerical noise when simulated and analyzed at different temperatures. The analysis temperature is a command-line option to the `ndfes` program (`--temp`); the default value is 298. Therefore, you should either: set all simulation temperatures to 298 or adjust all simulation temperatures to be the same value (T) and then set `--temp=`T when running `ndfes`.

- Column 3: *string*. A dumpave filename. The dumpave contains the raw samples obtained from biased sampling.

- Column 4: *float*. Reaction coordinate 1 harmonic biasing potential equilibrium position. The units must be consistent with the force constant.

- Column 5: *float*. Reaction coordinate 1 harmonic biasing potential prefactor. The biasing potential is defined as $U = f * (x - x_0)^2$ rather than $U = (k/2) * (x - x_0)^2$. The prefactor is half the force constant f, not the force constant k itself.

- Columns 6–7, 8–9, $\cdots$, *optional*. These are the harmonic potential positions and prefactors for the other reaction coordinates.

5.2 `ndfes` dumpave format

The reaction coordinates observed during biased sampling are saved in a dumpave file. Each line of the dumpave is a sample. The columns of the dumpave files are described below.

- Column 1: *integer*. The timestep. When `ndfes` reads the dumpave file, it will compare the timestep to the previous sample. If the timestep is the same, then the current sample will overwrite the previous sample. If they are different, then the current sample is treated as a new sample.
- Column 2–(N_{dim} + 1): *float*. The values of the N_{dim} reaction coordinates.
- Columns (N_{dim} + 2)–(N_{dim} + 2 + N_{ham}), *optional*: *float*. The unbiased potential energies of the N_{ham} Hamiltonians. These are only needed when evaluating the wTP or gwTP methods.

5.3 `ndfes` checkpoint file format

The `ndfes` output is stored in a XML–formatted checkpoint file.

- The XML root is a tag named `<ndfes>`.
- The `<ndfes>` root contains 1-or-more `<model>` tags.
- Each `<model>` tag must have a unique integer "idx" attribute starting from 0; e.g., `<model idx="0">`.
- A `<model>` tag has a `<type>`*string*`</type>` child. The string body is either MBAR or VFEP.
- If the model type is VFEP, then the `<model>` tag has a `<order>`*integer*`</order>` child. The vFEP method is solved using a Cardinal B-spline basis, and the integer value is the B-spline order.

- A <model> tag has a <grid> child.
- A <grid> tag has N_{dim} <dim> children.
- A <dim> tag must have a unique integer "idx" attribute starting from 0; e.g., <dim idx="0">.
- A <dim> tag has a <xmin>*float*</xmin> child. The float is the most negative (least positive) range of the regular grid in this dimension.
- A <dim> tag has a <xmax>*float*</xmax> child. The float is the least negative (most positive) range of the regular grid in this dimension.
- A <dim> tag has a <size>*integer*</size> child. The integer is the number of bins along the dimension. If the dimension is not periodic, then the width of a bin is $w = (x_{\text{max}} - x_{\text{min}})/(N_{\text{size}})$. If the dimension is periodic, then the width of a bin is $w = (x_{\text{max}} - x_{\text{min}})/(N_{\text{size}} - 1)$.
- A <dim> tag has a <isper>*integer*</isper> child. If the integer is 0, then the dimension is not periodic. If the integer is 1, then the dimension is periodic.
- A model has 1-or-more <bin> children.
- A <bin> tag must have a unique integer "idx" attribute; e.g., <bin idx="5">. The integer is the "global bin index" of the bin; a single integer that uniquely defines the position of a bin within the regular grid. It can be calculated from: $i + (j + (k + (\cdots) * N_k) * N_j$, where $i, j, k, \cdots$ are the 0-based "bin indexes" in each dimension, and $N_j, N_k, \cdots$ are the sizes of each dimension.
- A <bin> tag has N_{dim} <bidx idx="*integer*">*integer*</bidx> children. The attribute is a 0-based index of the dimension, and the body is the "bin index" within the dimension. The collection of bin indexes is an alternate way to uniquely identify a bin.
- A <bin> tag contains a <size>*integer*</size>. The integer is the number of samples within a bin.

- If the model type is MBAR, then the <bin> tag must also contain <val>*float*</val>, <err>*float*</err>, and <re>*float*</re> children. These are the free energy value and standard error (in kcal/mol). The RE value is the reweighting entropy (unitless).

- If the model type is VFEP, then the <model> tag must contain 2-or-more <corner idx="*integer*"> children, where the integer is a "global corner index". A "corner" is the separator that divides 2 bins in a direction. The global corner index is analogous to the global bin index, except that it uniquely identifies corners rather than bins.

- A <corner> tag contains <val>*float*</val>, <err>*float*</err> children. These are the B-spline parameters and uncertainties in kcal/mol.

5.4 ndfes-genbias metafile format

The ndfes-genbias program directly reads a "genbias metafile", which differs from the ndfes metafile format. Each line of the metafile characterizes a biased state and provides a filename that contains the raw samples.

- Column 1: *integer*. This is a zero-based integer, known as the "Hamiltonian index". The value of the integer should be 0 unless one is calculating the wTP or gwTP methods. In these cases, the dumpave file contains 2-or-more extra columns of unbiased potential energies, and the Hamiltonian index indicates which extra column corresponds to the unbiased potential energy of the sampled state.

- Column 2: *float*. The simulation temperature in Kelvin. The original intention of including the temperature within the metafile was to allow for the possibility of simulating biased states at different temperatures and then analyzing the free energy surface at another temperature. This functionality has not been extensively tested, and initial tests have suggested that the free energy surfaces suffer from significant numerical noise when simulated and analyzed at different temperatures. The analysis temperature is a command-line option to the ndfes

program (`--temp`); the default value is 298. Therefore, you should either: set all simulation temperatures to 298 or adjust all simulation temperatures to be the same value (T) and then set `--temp=`T when running `ndfes`.

- Column 3: *string*. A dumpave filename. The dumpave contains the raw samples obtained from biased sampling.

- Column 4: *integer*. This is a zero-based integer, known as the "bias index". The value of the integer identifies the column of bias potential energies within the "genbias dumpave" file. For example, a bias index of 0 means that the simulation was performed while subjecting the system to the first extra column of bias energies listed immediately after the reaction coordinates within the dumpave file. Unlike the metafiles used by `ndfes`, the `ndfes-genbias` metafiles cannot be easily concatenated to aggregate sampling, because there is absolutely no guarantee that they use the same set of biasing potentials or if their bias indexes are consistent with each other.

5.5 `ndfes-genbias` dumpave format

The reaction coordinates observed during biased sampling are saved in a dumpave file. Each line of the dumpave is a sample. The columns of the dumpave files are described below. Note that the dumpave file format used by `ndfes-genbias` is different than the format used by `ndfes`.

- Column 1: *integer*. The timestep. When `ndfes` reads the dumpave file, it will compare the timestep to the previous sample. If the timestep is the same, then the current sample will overwrite the previous sample. If they are different, then the current sample is treated as a new sample.

- Column 2–(N_{dim} + 1): *float*. The values of the N_{dim} reaction coordinates.

- Columns (N_{dim} + 2)–(N_{dim} + 2 + N_{bias}): *float*. The potential energy bias values of the N_{bias} bias functions. Note that these are the values of the "biasing potential" (W), not the "biased

potential energy” ($U + W$, where U and W refer to the unbiased potential energy and the biasing potential, respectively).

- Columns ($N_{\text{dim}} + 2 + N_{\text{bias}}$)–($N_{\text{dim}} + 2 + N_{\text{bias}} + N_{\text{ham}}$), *optional*: *float*. The unbiased potential energies of the N_{ham} Hamiltonians. These are only needed when evaluating the wTP or gwTP methods.

5.6 `edgembar` XML input

The `edgembar` program reads a XML input file. The contents of the file are defined below.

- The XML root tag is `<edge name="`*string*`">`. The string is of the form “A B” which denotes a transformation from state *A* to state *B*. These are often the names of ligands. Ligand names must not contain a tilde or whitespace characters.

- The `<edge>` tag contains one or two `<env name="`*string*`">` children. If there is only 1 environment, then the name must be “target”. If there are 2 environments, one must by “target” and the other must be “reference”.

- A `<env>` tag must contain 1-or-more `<stage name="`*string*`">` children. The stage can be named anything descriptive.

- A `<stage>` tag must contain 1-or-more `<trial name="`*string*`">` children. The trial can be named anything descriptive; however, it is usually an integer (1) or a “t” followed by an integer (t1).

- A `<trial>` tag must contain a `<dir>path/to/efep/files</dir>` child. The text is a filename path to a directory which contains files named “efep_*tlam*_*elam*.dat”. These are the potential energies of state *elam* (the energy lambda) evaluated at the samples generated from simulation of state *tlam* (the trajectory lambda).

- The `<trial>` tag must contain 2-or-more `<ene>{\em string}</ene>` children. The string is usually a number between 0 and 1 that identifies a state by a λ free energy coordinate. The

ene strings listed within a trial are grouped into pairs and the raw data files expected to be named `dir`/efep_`tlam`_`elam`.dat where tlam and elam are combinations of ene strings.

5.7 `edgembar` raw data files

The `edgembar` program analyzes energies stored in "efep" files named efep_*tlam*_*elam*.dat, where *tlam* is a string that denotes the state that produced the trajectory (the trajectory lambda) and *elam* is a string that denotes the state that produced the energy stored within the file (the energy lambda). Each line of an efep file corresponds to a sample produced by state tlam. There are two columns.

- Column 1: The simulation time (ps).
- Column 2: The potential energy of state *elam* (kcal/mol).

6 Examples

6.1 1-dimensional FES calculated from vFEP and MBAR

`ndfes` requires dumpave files and a metafile to calculate a free energy surface. The following scenario calculates a 1-dimensional free energy surface with vFEP and MBAR. Here is the general layout.

```
[user@computer] ls
     dumpaves/  t01.metafile  t02.metafile  t03.metafile  t04.metafile
[user@computer] ls dumpaves/
     t01/  t02/  t03/  t04/
[user@computer] ls dumpavess/t01/
     win_0.00.dumpave   win_-0.10.dumpave  win_0.10.dumpave
     [etc...]
```

The dumpave files contain the timestep and reaction coordinate value. In this example, the reaction coordinate values are Å, but the units are not important as long as they are consistent with the harmonic potential definition within the metafile.

```
[user@computer] head -n 3 dumpavess/t01/win_0.00.dumpave
       0        0.046000
      50        0.106000
     100        0.040000
```

The metafile lists the biased states. The last 2 columns are the position and prefactor for the harmonic potential.

```
[user@computer] head -n 3 t01.metafile
0 298.00 dumpaves/t01/win_-1.30.dumpave   -1.300000    1.50000000000000e+02
0 298.00 dumpaves/t01/win_-1.20.dumpave   -1.200000    1.50000000000000e+02
0 298.00 dumpaves/t01/win_-1.10.dumpave   -1.100000    1.50000000000000e+02
```

The following calculates the free energy surface with vFEP using 5th order Cardinal B-splines and a 0.15 Å regular grid. The bin width units are assumed to be the same as the units within the dumpave file. The errors are estimated from cyclic moving block bootstrap analysis using 50 bootstrap samples. The output is written to t01.vfep.chk

```
[user@computer] ndfes --vfep -o 5 -w 0.15 --nboot=50 \
                    -c t01.vfep.chk t01.metafile
```

This similarly performs analysis with MBAR.

```
[user@computer] ndfes --mbar -w 0.15 --nboot=50 -c t01.mbar.chk t01.metafile
```

One can print the free energy values with `ndfes-PrintFES.py`.

```
[user@computer] ndfes-PrintFES.py t01.mbar.chk > t01.mbar.dat
[user@computer] head -n 3 t01.mbar.dat
```

```
-1.42500000    4.050012e+00    3.018e-01    0.543     125
-1.27500000    3.536166e+00    9.646e-02    0.994    2414
-1.12500000    2.545974e+00    8.460e-02    0.988    3080
```

The first column is the location of a bin center. The second column is the free energy (kcal/mol). The third column is the standard error (kcal/mol). The fourth column is the reweighting entropy. The last column is the number of samples within the bin. If this was a 2-dimensional FES, then the first two columns would specify the location of a bin center, for example.

6.2 Analyzing aggregate sampling and updating errors to account for the variation between trials

One can separately analyze independent simulation trials, for example.

```
[user@computer] ndfes --mbar -w 0.15 --nboot=50 -c t01.chk t01.metafile
[user@computer] ndfes --mbar -w 0.15 --nboot=50 -c t02.chk t02.metafile
[user@computer] ndfes --mbar -w 0.15 --nboot=50 -c t03.chk t03.metafile
[user@computer] ndfes --mbar -w 0.15 --nboot=50 -c t04.chk t04.metafile
```

A best estimate can be made by aggregating the samples and analyzing everything.

```
[user@computer] ndfes-CombineMetafiles.py t01.metafile t02.metafile \
                  t03.metafile t04.metafile -o all.metafile
[user@computer] ndfes --mbar -w 0.15 --nboot=50 -c all.chk all.metafile
```

One can write a new checkpoint file, new.chk, that has the same free energy values as all.chk, but whose error estimates account for the variation between the independent trials.

```
[user@computer] ndfes-AvgFESs.py --ene all.chk --fes t01.chk --fes t02.chk \
                  --fes t03.chk --fes t04.chk --out new.chk
```

One can print the free energy values with `ndfes-PrintFES.py`.

```
[user@computer] ndfes-PrintFES.py new.chk > new.dat
```

6.3 2-dimensional FES calculated from MBAR

The main difference between a 2-dimensional FES in comparison to the 1-dimensional example discussed in the previous sections is: 1. the metafile contains 2 extra columns (the position and prefactor of the 2nd harmonic potential), and 2. the dumpave files contain 1 extra column (the observed values of the 2nd reaction coordinate).

```
[user@computer] head -n 3 twod.metafile
0 298.00 dumpaves/it06/img01.dumpave  -0.857348  5.0e+01  -2.020608  5.0e+01
0 298.00 dumpaves/it06/img02.dumpave  -0.685935  5.0e+01  -1.922061  5.0e+01
0 298.00 dumpaves/it06/img03.dumpave  -0.468861  5.0e+01  -1.867230  5.0e+01
```

```
[user@computer] head -n 3 dumpaves/it06/img01.dumpave
       0       -0.784000       -2.108000
      10       -0.847000       -2.042000
      20       -0.828000       -2.045000
```

One can calculate the FES and store it in twod.chk. In this version, the regular grid bin widths in each dimension is 0.15 Å.

```
[user@computer] ndfes --mbar -w 0.15 --nboot=50 -c twod.chk twod.metafile
```

We can use the `-w` option multiple times to specify different bin widths in each dimension. The following sets a bin width of 0.1 Å for the first reaction coordinate and a bin width of 0.3 Å for the second reaction coordinate.

```
[user@computer] ndfes --mbar -w 0.1 -w 0.3 --nboot=50 \
                     -c twod.chk twod.metafile
```

One can print the free energy values with `ndfes-PrintFES.py`.

```
[user@computer] ndfes-PrintFES.py twod.chk > twod.dat
[user@computer] head -n 3 twod.dat
```

```
-0.97500000    -2.17500000    2.332242e+01    4.525e-01    0.833     10
-0.82500000    -2.17500000    2.312587e+01    3.394e-01    0.880     38
-0.67500000    -2.17500000    2.401594e+01    4.349e-01    0.922      3
```

The first two columns are the coordinates of a bin center. The remaining columns are the free energy (kcal/mol), standard error (kcal/mol), reweighting entropy, and the number of samples in the bin.

6.4 Calculation of an alchemical transformation free energy

The `edgembar` input is an XML file. An example is shown below.

```
[user@computer] cat xml/1h1r~1h1s.xml
<edge name="1h1r~1h1s">
        <env name="target">
                <stage name="STAGE">
                        <trial name="t1">
                                <dir>dats/1h1r~1h1s/com/t1</dir>
                                <ene>0.00000000</ene>
                                <ene>0.08333333</ene>
                                <ene>0.16666667</ene>
                                <ene>0.25000000</ene>
                                <ene>0.33333333</ene>
                                <ene>0.41666667</ene>
                                <ene>0.50000000</ene>
                                <ene>0.58333333</ene>
                                <ene>0.66666667</ene>
                                <ene>0.75000000</ene>
                                <ene>0.83333333</ene>
```

```
                <ene>0.91666667</ene>
                <ene>1.00000000</ene>
            </trial>
            <trial name="t2">
                <dir>dats/1h1r~1h1s/com/t2</dir>
                <ene>0.00000000</ene>
                <ene>0.08333333</ene>
                <ene>0.16666667</ene>
                <ene>0.25000000</ene>
                <ene>0.33333333</ene>
                <ene>0.41666667</ene>
                <ene>0.50000000</ene>
                <ene>0.58333333</ene>
                <ene>0.66666667</ene>
                <ene>0.75000000</ene>
                <ene>0.83333333</ene>
                <ene>0.91666667</ene>
                <ene>1.00000000</ene>
            </trial>
           [...ETC...]
        </stage>
</env>
<env name="reference">
        <stage name="STAGE">
            <trial name="t1">
                <dir>dats/1h1r~1h1s/aq/t1</dir>
                <ene>0.00000000</ene>
```

```
                    [...ETC...]
                </trial>
            </stage>
        </env>
</edge>
```

The specified directories are expected to have the raw data ("efep files") for the given edge, environment, stage, and trial.

```
[user@computer] ls dats/1h1r~1h1s/com/t1
dvdl_0.00000000.dat             efep_0.50000000_0.08333333.dat
dvdl_0.08333333.dat             efep_0.50000000_0.16666667.dat
dvdl_0.16666667.dat             efep_0.50000000_0.25000000.dat
dvdl_0.25000000.dat             efep_0.50000000_0.33333333.dat
dvdl_0.33333333.dat             efep_0.50000000_0.41666667.dat
dvdl_0.41666667.dat             efep_0.50000000_0.50000000.dat
dvdl_0.50000000.dat             efep_0.50000000_0.58333333.dat
dvdl_0.58333333.dat             efep_0.50000000_0.66666667.dat
dvdl_0.66666667.dat             efep_0.50000000_0.75000000.dat
dvdl_0.75000000.dat             efep_0.50000000_0.83333333.dat
dvdl_0.83333333.dat             efep_0.50000000_0.91666667.dat
dvdl_0.91666667.dat             efep_0.50000000_1.00000000.dat
dvdl_1.00000000.dat             efep_0.58333333_0.00000000.dat
efep_0.00000000_0.00000000.dat  efep_0.58333333_0.08333333.dat
efep_0.00000000_0.08333333.dat  efep_0.58333333_0.16666667.dat
efep_0.00000000_0.16666667.dat  efep_0.58333333_0.25000000.dat
efep_0.00000000_0.25000000.dat  efep_0.58333333_0.33333333.dat
[...ETC...]
efep_0.41666667_0.83333333.dat  efep_1.00000000_0.91666667.dat
```

```
efep_0.41666667_0.91666667.dat  efep_1.00000000_1.00000000.dat
efep_0.41666667_1.00000000.dat  rem.log.yaml
efep_0.50000000_0.00000000.dat
```

The “dvdl” and “rem.log.yaml” files are optional. If one wanted to perform a BAR calculation rather than a MBAR calculation, the the full matrix of efep files is not necessary. To perform the calculation, run `edgembar`. The output is a python script. The name replaces the “.xml” suffix with “.py”.

```
[user@computer] edgembar --fwdrev --halves xml/1h1r~1h1s.xml
[user@computer] ls xml/1h1r~1h1s.py
```

The `--fwdrev --halves` options tell `edgembar` to perform the calculation many times to create a timeseries analysis. The `fwdrev` timeseries analysis compares the free energies calculated from the first X% of the simulation data to the those calculated from the last X% of data. The `halves` analysis excludes X% of the simulation from the start as equilibration, splits the remaining 100-X% of data into two halves, and then compares the free energies calculated from the first and second half.

To view a brief summary of the results printed to stdout, use the `--brief` option.

```
[user@computer] python3 ./xml/1h1r~1h1s.py --brief
           Edge        Env       Stage      Trial    Energy    Error
      1h1r~1h1s                                      -1.539    0.264
      1h1r~1h1s     target                         -164.457    0.201
      1h1r~1h1s     target       STAGE             -164.457    0.201
      1h1r~1h1s     target       STAGE         t1  -164.164    0.221
      1h1r~1h1s     target       STAGE         t2  -164.673    0.247
      1h1r~1h1s     target       STAGE         t3  -164.534    0.211
      1h1r~1h1s  reference                         -162.918    0.172
      1h1r~1h1s  reference       STAGE             -162.918    0.172
```

```
1h1r~1h1s   reference      STAGE          t1   -162.900    0.290
1h1r~1h1s   reference      STAGE          t2   -162.742    0.201
1h1r~1h1s   reference      STAGE          t3   -163.112    0.193
```

The $\Delta\Delta G$ is -1.539 ± 0.264 kcal/mol, where the "error" is a standard error. The ΔG in the target environment is -164.457 ± 0.201 kcal/mol. The ΔG in the reference environment is -162.918 ± 0.172 kcal/mol. The remaining lines breakdown the ΔG values into stages and trials within a stage.

To view a HTML–formatted "edge report", simply run the python output without any arguments.

```
[user@computer] python3 ./xml/1h1r~1h1s.py
[user@computer] ls ./xml/1h1r-1h1s.html
```

Images of the edge report are shown below.

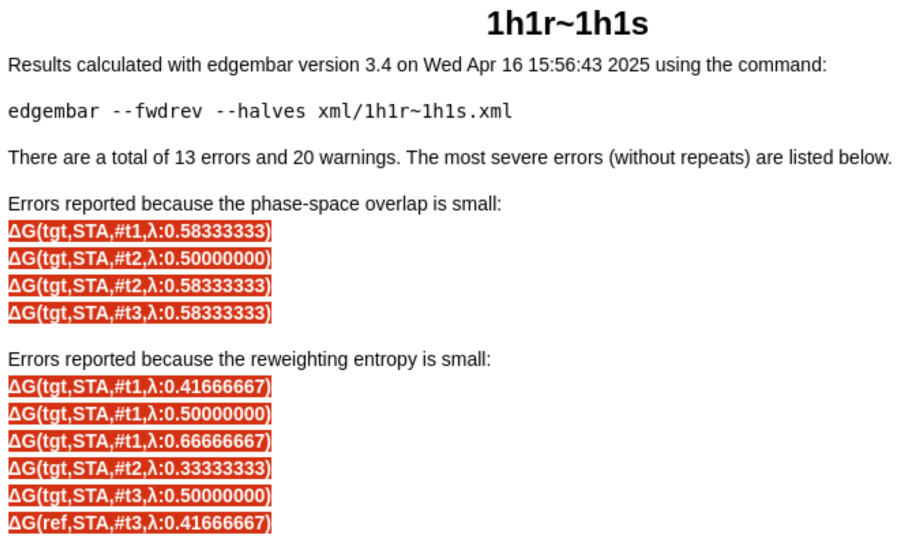

Figure S1: The top of the edge report shows the edge, date, command line options, and a summary of potential issues with the data that the user should be aware of.

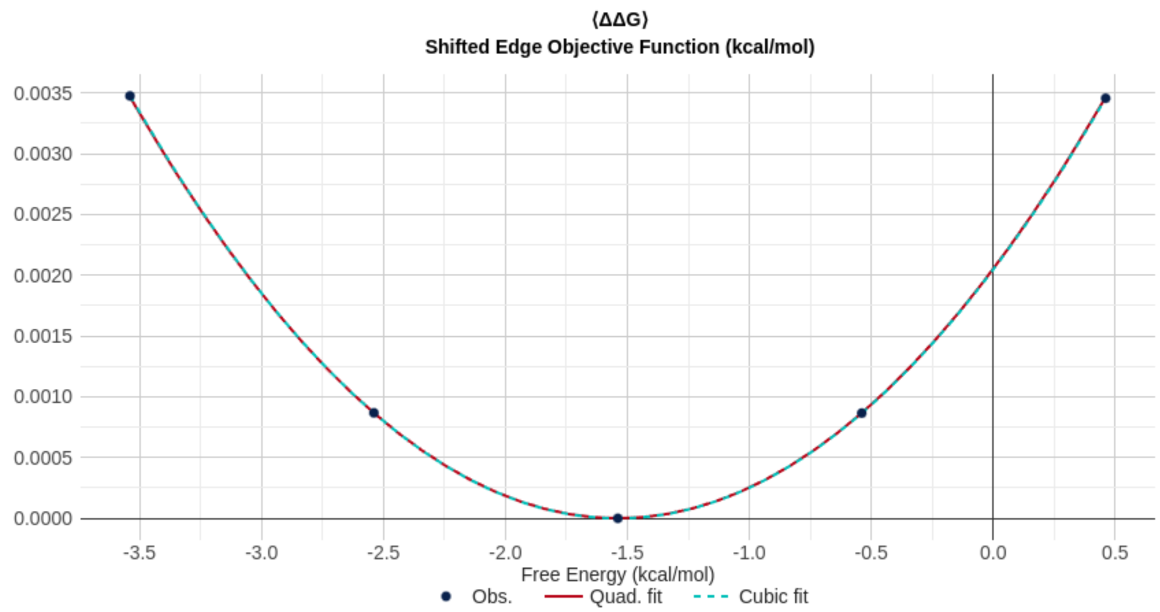

The objective function polynomial fit is of the form:

F(x) = c0 + c2*(x-x0)^2 + c3*(x-x0)^3

where x0=-1.5391284325e+00 c0=1.1911645458e+00 c2=8.6641528744e-04 c3=-1.2016460845e-06. The plot excludes the c0 term.

A cubic fit produces a Pearson correlation of 1.00000.

A quadratic fit produces a Pearson correlation of 0.99999.

Figure S2: The figure shows the edge's effective objective function obtained from a series of constrained solutions. The x-axis is the $\Delta\Delta G$ and the y-axis is the MBAR objective function value. The lines are a quadratic and cubic fits.

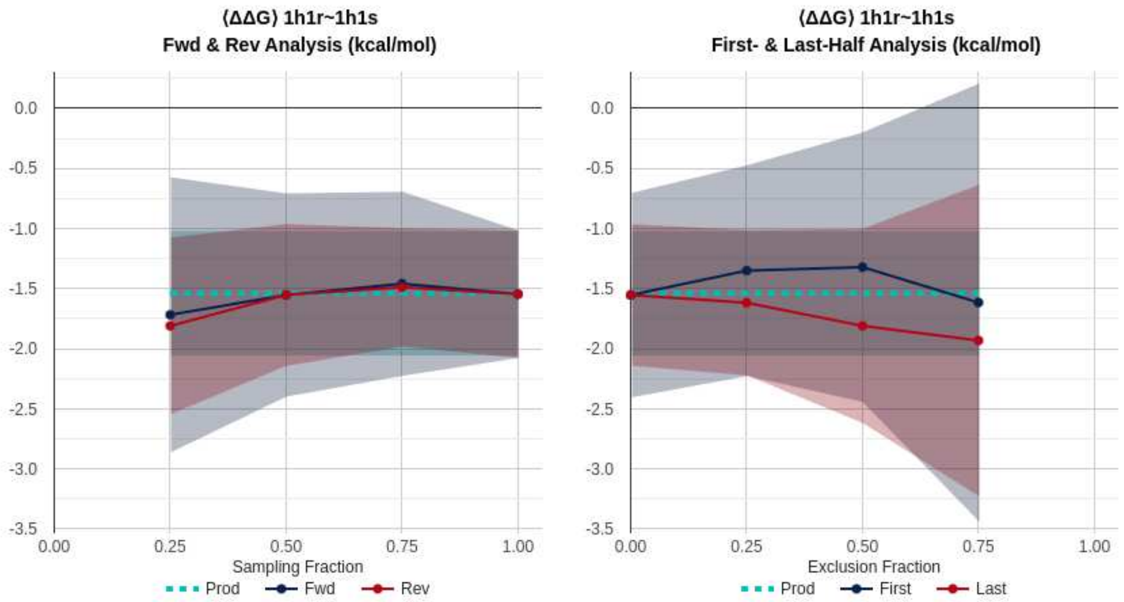

The relative free energy ⟨ΔΔG⟩ is the difference in complexed and solvated environments:

⟨ΔΔG⟩ = ⟨ΔG(tgt,STA)⟩ - ⟨ΔG(ref,STA)⟩

Click here to see ⟨ΔG(tgt,STA)⟩

Click here to see ⟨ΔG(ref,STA)⟩

Decomposition of ⟨ΔΔG⟩ into environmental ΔG values

Sampling	⟨ΔΔG⟩	⟨ΔG(tgt)⟩	⟨ΔG(ref)⟩	⟨f_{eq}⟩	⟨g_{prod}⟩
Prod.	-1.539 ± 0.264	-164.457 ± 0.201	-162.918 ± 0.172	0.01	1
75% Eq.	-1.810 ± 0.374	-164.608 ± 0.297	-162.798 ± 0.228	0.75	
50% Eq.	-1.553 ± 0.301	-164.587 ± 0.224	-163.034 ± 0.201	0.50	
25% Eq.	-1.489 ± 0.251	-164.357 ± 0.169	-162.868 ± 0.186	0.25	
0% Eq.	-1.546 ± 0.271	-164.471 ± 0.217	-162.925 ± 0.162	0.00	

Comparison of ⟨ΔΔG⟩ MBAR and TI results

Method	ΔΔG	ΔΔG-MBAR	T
Linear Interp.	-1.469 ± 0.266	0.070 ± 0.375	0.187
Natural Cubic	-1.470 ± 0.267	0.069 ± 0.375	0.185
Clamped Cubic	-1.469 ± 0.266	0.070 ± 0.375	0.187
MBAR	-1.539 ± 0.264		

Figure S3: The $\Delta\Delta G$ is decomposed into environmental ΔG values. Energies are shown in kcal/mol. The f_{eq} column shows how much data was excluded from the beginning of the simulations, on average. The g_{prod} column is the statistical inefficiency of the analyzed region. The "Prod." row analyzes the production sampling after automagic identification of the unequilibrated data. The remaining rows manually exclude a specific percentage of the simulations as equilibration. The plots show the timeseries analysis of the $\Delta\Delta G$. The shaded areas are 95% confidence intervals (1.96*standard error). The table at the bottom compares the MBAR result to TI calculations (if the dvdl files were found in the directory along side the efep files). The TI integration is performed with the trapezoidal rule and integration of clamped and natural cubic splines. The $\Delta\Delta G$-MBAR shows the absolute difference between the TI and MBAR free energies. The "T" column is the *t-statistic*: a measure of difference in units of standard error.

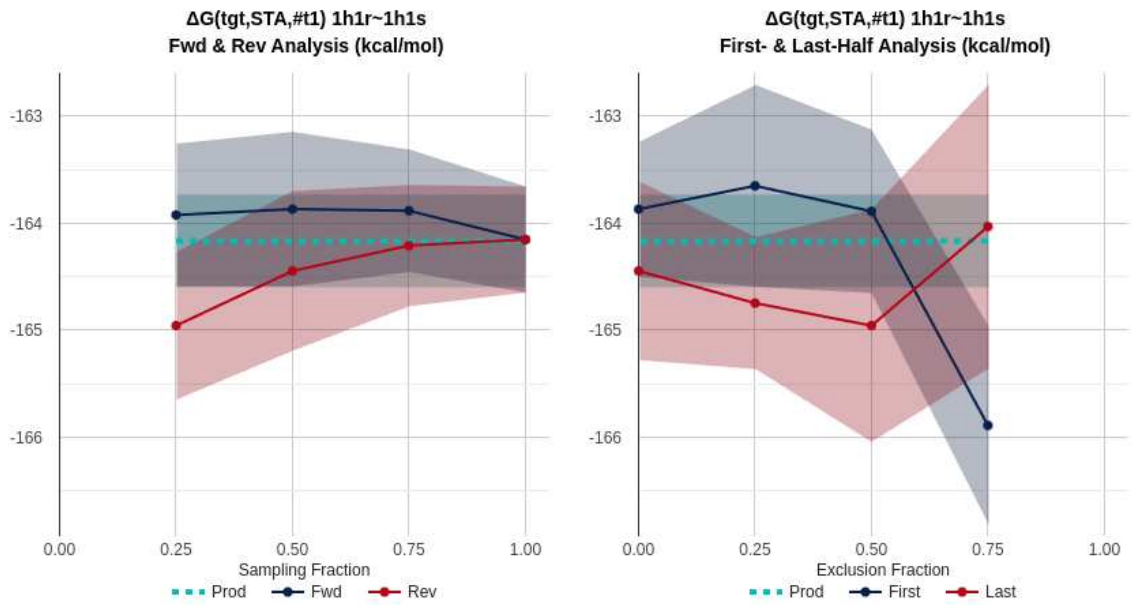

The free energy ΔG(tgt,STA,#t1) was used in the calculation of the following quantities:
Click here to see ⟨ΔΔG⟩
Click here to see ⟨ΔG(tgt,STA)⟩

ΔG(tgt,STA,#t1)

Traj	ΔG	Prod. Region					All Data		S	RE		Conv?
	MBAR	f_{eq}	N_{eq}	g_{prod}	g_{ana}	N_{ana}	g	N	Fwd	Fwd	Rev	
0.00000000	0.000 ± 0.000	0.00	0	1	1	250	1	250	0.98	1.00		
0.08333333	-0.661 ± 0.002	0.00	0	2	2	250	2	250	0.81	0.97	1.00	
0.16666667	-4.668 ± 0.013	0.00	0	1	1	250	1	250	0.61	0.84	0.97	
0.25000000	-13.907 ± 0.032	0.00	0	1	1	250	1	250	0.49	0.78	0.86	
0.33333333	-28.904 ± 0.051	0.00	0	1	1	250	1	250	0.30	0.53	0.73	
0.41666667	-48.850 ± 0.095	0.00	0	1	1	250	1	250	0.17	0.30	0.72	
0.50000000	-72.950 ± 0.147	0.00	0	2	2	250	2	250	0.19	0.55	0.35	
0.58333333	-98.661 ± 0.187	0.00	0	1	1	250	1	250	0.13	0.49	0.14	
0.66666667	-122.925 ± 0.223	0.21	52	3	3	198	3	250	0.32	0.61	0.34	
0.75000000	-143.109 ± 0.226	0.00	0	1	1	250	1	250	0.43	0.80	0.70	
0.83333333	-156.740 ± 0.223	0.00	0	1	1	250	1	250	0.86	0.96	0.85	
0.91666667	-163.085 ± 0.221	0.00	0	1	1	250	1	250	0.94	1.00	0.95	
1.00000000	-164.164 ± 0.221	0.00	0	1	1	250	1	250			1.00	

Figure S4: The plots show the timeseries analysis of a specific trial. The table lists the alchemical states, their free energies, and standard errors (where the first state defines the zero of free energy). The columns under "Prod Region" are statistics gathered from the "production region" after discarding some fraction of simulation from the beginning, as determined from the detection algorithm. $f_{\rm eq}$ is the fraction of the simulation discarded as equilibration. $N_{\rm eq}$ is the number of samples excluded as equilibration. $g_{\rm prod}$ is the statistical inefficiency of the remaining samples after discarding the equilibration. The columns under "All Data" similarly list the statistical inefficiency and the total number of samples from the entire simulation (without discarding anything). The $g_{\rm ana}$ and $N_{\rm ana}$ are the statistical inefficiency and number of samples of the analyzed data. By default, the analyzed data *is* the production region after discarding the equilibration; however, one can run `edgembar` with the `-no-auto` option to skip the detection algorithm. The "S" columns is the phase space overlap metric. The "Fwd RE" and "Rev RE" are the forward and reverse reweighting entropies. One can reweight the samples of λ_i to try and mimic the distribution of λ_{i+1}. If the weights are uniform, then the RE is 1.0. If only a few samples contribute a significant weight, then the RE is close to 0.0. In other words, it is another measure of how well adjacent states overlap. The reverse RE similarly compares the distribution of λ_i to λ_{i-1}.

6.5 Networkwide analysis of alchemical free energies

To perform networkwide analysis, run edgembar on each edge in the graph. You should then have a python output file for each edge. Use edgembar-WriteGraphHtml.py to perform the analysis and write a "graph report".

```
[user@computer] edgembar-WriteGraphHtml.py xml/1h1r~1h1s.py  \
                    xml/1h1r~1oiu.py  xml/1h1s~1h1q.py \
                    xml/1h1s~1oiu.py  xml/1oiu~1h1q.py \
                    -o xml/graph.html
[...or...]
[user@computer] edgembar-WriteGraphHtml.py $(ls xml/*~*.py) \
                    -o xml/graph.html
```

Images of graph.html are shown below. Note that all of the FE-ToolKit programs and scripts include a --help option that gives a summary of the available options. The edgembar-WriteGraphHtml.py script includes 3 important options: --refnode=NAME, --expt=FILENAME, and --constrain=LIGA~LIGB. The --refnode option allows one to manually choose the ligand defining the zero of energy in the network analysis. The --expt=FILENAME option allows one to read a list of experimental (or reference) ligand free energies. The file contains one row per ligand. Each row contains 2 columns. The first column is the name of a ligand. The second column is the experimental (or reference) free energy (in kcal/mol). If this file is present, then the graph report will display extra columns of experimental (or reference) ligand and edge free energies. The --contrain option allows one to constrain the free energy of an edge to match the difference in ligand free energies listed in the file of experimental (or reference) free energies. One can use the --contrain option multiple times to constrain more than 1 edge. The constrained free energy does not have to be connected through alchemical transformations; the constraint applies to a difference in ligand free energies regardless of whether they are directly connected by an edge.

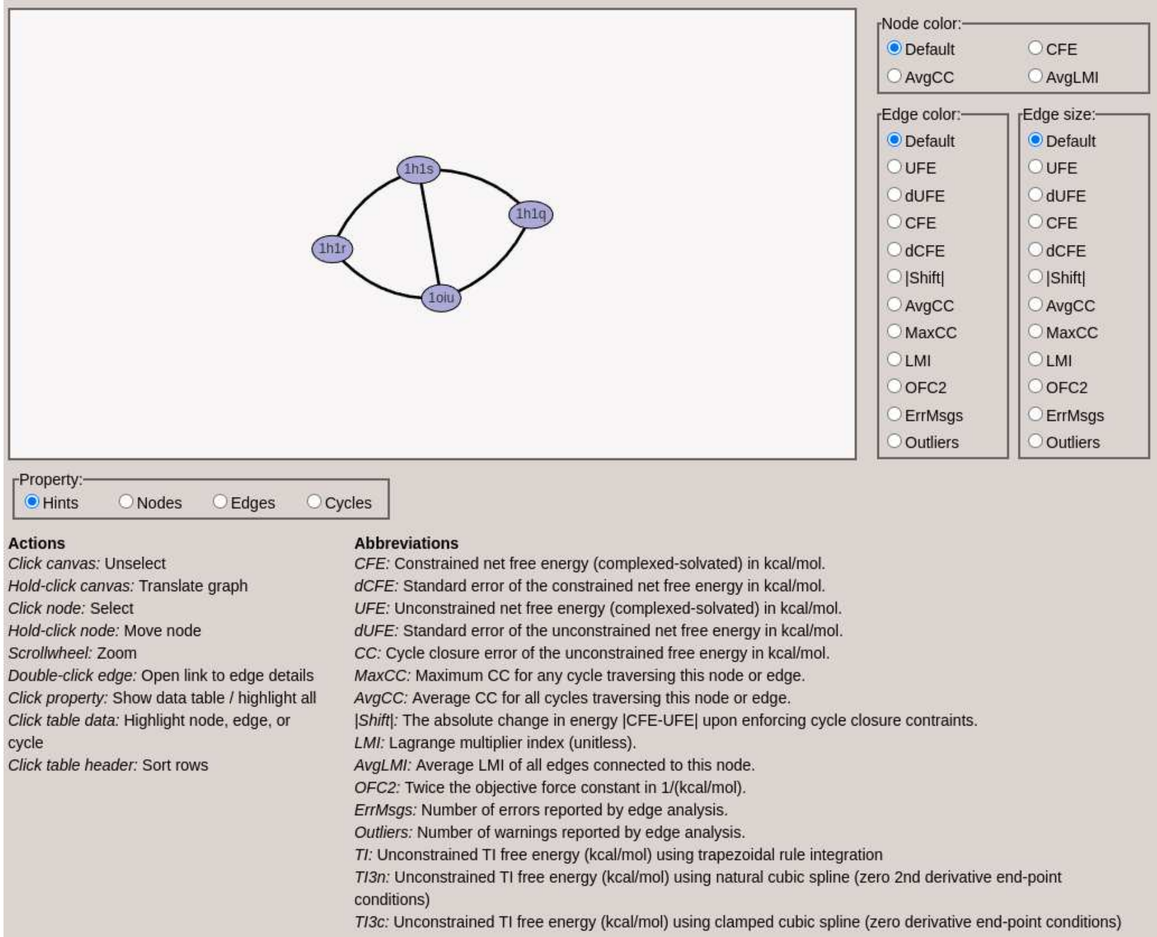

Figure S5: The top of the HTML graph report displays the network. The network can be dynamically manipulated with the mouse using the mouse actions described below the Property toggle. The node and edge colors and edge widths can be manipulated with the toggles shown on the right. Blue and red represent most negative (least positive) and most positive (least negative) values, respectively. The tables refer to properties based on abbreviations. See section 2.3 for more information. To view the tables of node, edge, and cycle closure properties, select the appropriate option from the Property toggle.

Property: ○ Hints ◉ Nodes ○ Edges ○ Cycles

Node ↑	CFE ↑	dCFE ↑	LMI ↑	AvgCC ↑
1h1q	0.000	0.166	5.64e-04	0.569
1h1r	-0.276	0.171	5.00e-05	0.403
1h1s	-1.834	0.133	6.28e-04	0.538
1oiu	-2.728	0.133	6.20e-04	0.538
**N**	**4**	**4**	**4**	**4**
**Mean**	**-1.209**	**0.151**	**4.65e-04**	**0.512**
**MeanAbs**	**1.209**	**0.151**	**4.65e-04**	**0.512**
**StdDev**	**1.295**	**0.021**	**2.78e-04**	**0.074**
**Min**	**-2.728**	**0.133**	**5.00e-05**	**0.403**
**Max**	**0.000**	**0.171**	**6.28e-04**	**0.569**

Figure S6: The Node Properties table lists the ligand free energies calculated from networkwide analysis. The CFE and dCFE values are the ligand free energies and standard errors. One of the ligands defines the zero of energy. LMI is the Lagrange multiplier index, and AvgCC is the average cycle closure error for all closed cycles that traverse the node. One can sort the columns by clicking the table header. One can highlight a node in the network display by clicking on a row. The bottom of the table shows basic statistics for the numbers shown in the column. These are the number of items, the mean value, mean absolute value, standard deviation, minimum, and maximum values.

Property: ○ Hints ○ Nodes ◉ Edges ○ Cycles

Edge ↑	UFE ↑	dUFE ↑	CFE ↑	dCFE ↑	TI ↑	TI3n ↑	TI3c ↑	Shift ↑	LMI ↑	OFC2 ↑	AvgCC ↑	MaxCC ↑	ErrMsgs ↑	Outliers ↑
1h1r~1h1s	-1.539	0.284	-1.558	0.216	-1.469	-1.470	-1.469	0.019	5.01e-05	8.66e-04	0.403	0.476	13	0
1h1r~1oiu	-2.471	0.290	-2.452	0.216	-2.591	-2.589	-2.591	0.019	4.98e-05	8.76e-04	0.403	0.476	4	0
1h1s~1h1q	1.642	0.275	1.834	0.213	1.745	1.747	1.745	0.192	5.75e-04	1.00e-03	0.569	0.807	3	0
1h1s~1oiu	-0.456	0.329	-0.894	0.188	-0.181	-0.180	-0.181	0.438	1.26e-03	4.76e-04	0.641	0.807	22	0
1oiu~1h1q	2.905	0.329	2.728	0.213	2.968	2.969	2.968	0.177	5.53e-04	1.09e-03	0.569	0.807	2	0
**N**	**5**	**5**	**5**	**5**	**5**	**5**	**5**	**5**	**5**	**5**	**5**	**5**	**5**	**5**
**Mean**	**0.016**	**0.301**	**-0.068**	**0.209**	**0.095**	**0.095**	**0.095**	**0.169**	**4.97e-04**	**8.62e-04**	**0.517**	**0.674**	**8**	**0**
**MeanAbs**	**1.802**	**0.301**	**1.893**	**0.209**	**1.791**	**1.791**	**1.791**	**0.169**	**4.97e-04**	**8.62e-04**	**0.517**	**0.674**	**8**	**0**
**StdDev**	**2.226**	**0.026**	**2.237**	**0.012**	**2.276**	**2.276**	**2.276**	**0.172**	**4.97e-04**	**2.35e-04**	**0.108**	**0.181**	**8**	**0**
**Min**	**-2.471**	**0.275**	**-2.452**	**0.188**	**-2.591**	**-2.589**	**-2.591**	**0.019**	**4.98e-05**	**4.76e-04**	**0.403**	**0.476**	**2**	**0**
**Max**	**2.905**	**0.329**	**2.728**	**0.216**	**2.968**	**2.969**	**2.968**	**0.438**	**1.26e-03**	**1.09e-03**	**0.641**	**0.807**	**22**	**0**

Figure S7: The Edge Properties table lists the edge free energies and standard errors calculated from isolated (UFE and dUFE) and networkwide (CFE and dCFE) analysis. If edges were analyzed with "dvdl" data files, then the thermodynamic integration results will also appear as extra columns. Clicking on a row will highlight an edge in the network display, and clicking on the edge name will open the HTML edge report in a new window or tab.

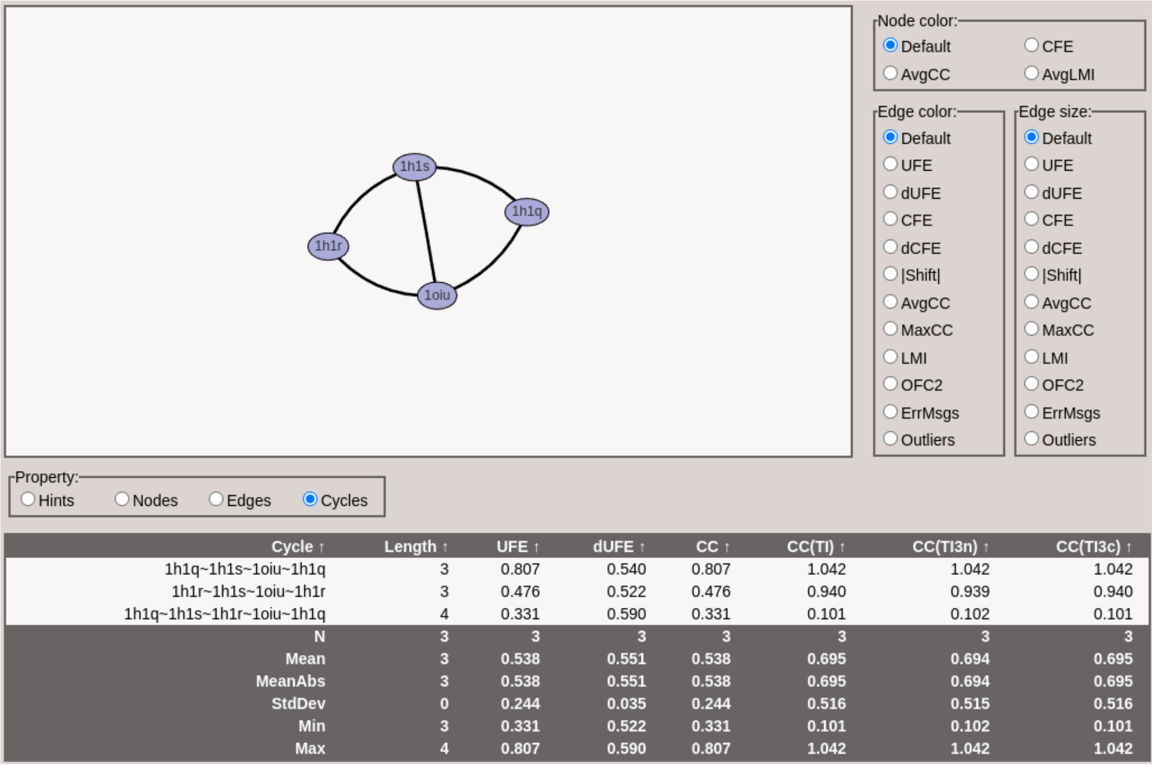

Cycle ↑	Length ↑	UFE ↑	dUFE ↑	CC ↑	CC(TI) ↑	CC(TI3n) ↑	CC(TI3c) ↑
1h1q~1h1s~1oiu~1h1q	3	0.807	0.540	0.807	1.042	1.042	1.042
1h1r~1h1s~1oiu~1h1r	3	0.476	0.522	0.476	0.940	0.939	0.940
1h1q~1h1s~1h1r~1oiu~1h1q	4	0.331	0.590	0.331	0.101	0.102	0.101
N	3	3	3	3	3	3	3
Mean	3	0.538	0.551	0.538	0.695	0.694	0.695
MeanAbs	3	0.538	0.551	0.538	0.695	0.694	0.695
StdDev	0	0.244	0.035	0.244	0.516	0.515	0.516
Min	3	0.331	0.522	0.331	0.101	0.102	0.101
Max	4	0.807	0.590	0.807	1.042	1.042	1.042

Figure S8: The Cycle Properties table lists the cycle closure errors. Clicking on a row will highlight the cycle in the network display.

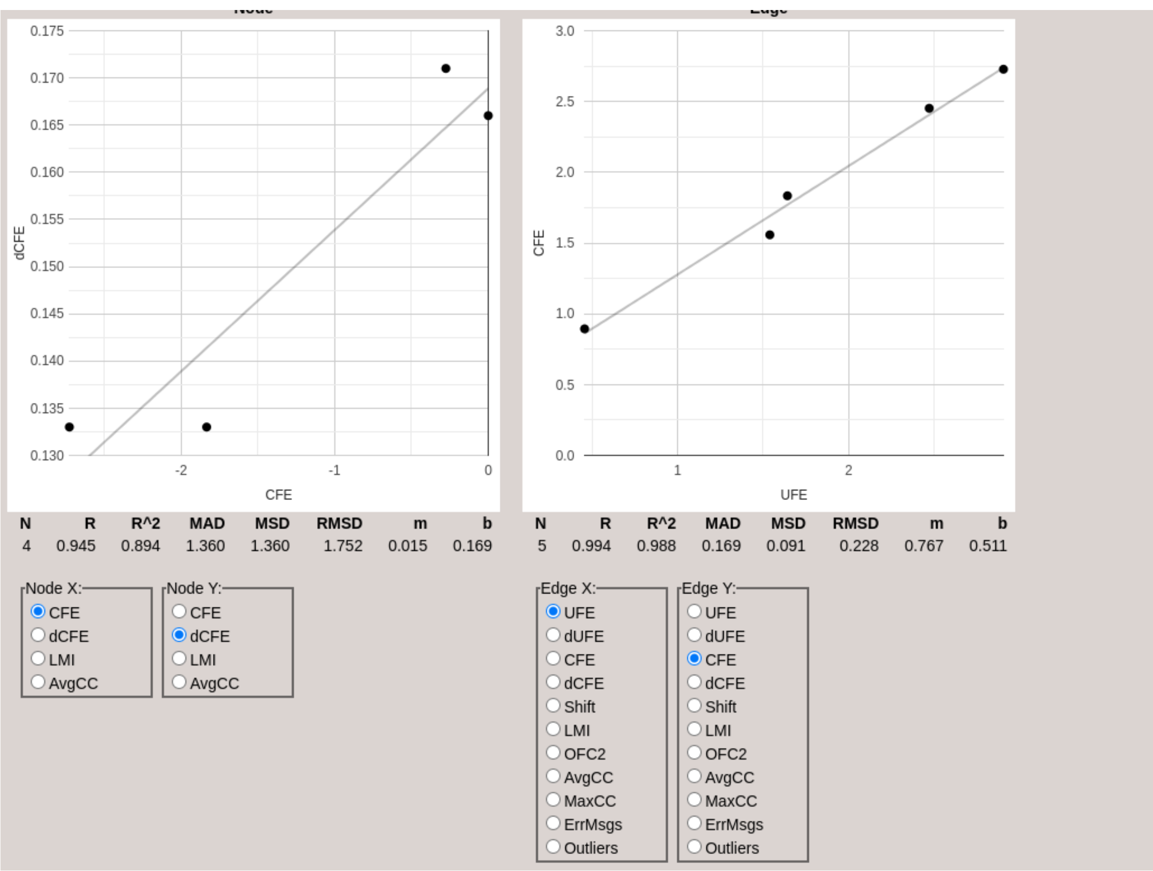

Figure S9: The bottom of the HTML graph report will show plots for the node and edge properties. You can independently choose properties for the X- and Y-axis to perform a linear regression. The R value is the correlation coefficient. The m and b values are the slope and intercept. MAD, MSD, and RMSD are the mean absolute deviation, mean signed deviation, and root mean squared deviation, respectively.

References

(S1) Grossfield, A. WHAM: the weighted histogram analysis method [Online], version 2.1.0; http://membrane.urmc.rochester.edu/wordpress/?page_id=126 (accessed April 2025).

(S2) Li, P.; Jia, X.; Pan, X.; Shao, Y.; Mei, Y. Accelerated Computation of Free Energy Profile at ab Initio Quantum Mechanical/Molecular Mechanics Accuracy via a Semi-Empirical Reference Potential. I. Weighted Thermodynamics Perturbation. *J. Chem. Theory Comput.* **2018**, *14*, 5583–5596.

(S3) Giese, T. J.; Zeng, J.; York, D. M. Multireference Generalization of the Weighted Thermodynamic Perturbation Method. *J. Phys. Chem. A* **2022**, *126*, 8519–8533.

(S4) Giese, T. J.; Ekesan, Ş.; McCarthy, E.; Tao, Y.; York, D. M. Surface-Accelerated String Method for Locating Minimum Free Energy Paths. *J. Chem. Theory Comput.* **2024**, *20*, 2058–2073.

(S5) Klimovich, P. V.; Shirts, M. R.; Mobley, D. L. Guidelines for the analysis of free energy calculations. *J. Comput.-Aided Mol. Des.* **2015**, *29*, 397–411.

(S6) Mezei, M. Polynomial path for the calculation of liquid state free energies from computer simulations tested on liquid water. *J. Comput. Chem.* **1992**, *13*, 651–656.

(S7) Resat, H.; Mezei, M. Studies on free energy calculations. I. Thermodynamic integration using a polynomial path. *J. Chem. Phys.* **1993**, *99*, 6052–6061.

(S8) Simonson, T. Free energy of particle insertion. *Mol. Phys.* **1993**, *80*, 441–447.

(S9) Beutler, T. C.; Mark, A. E.; van Schaik, R. C.; Gerber, P. R.; van Gunsteren, W. F. Avoiding singularities and numerical instabilities in free energy calculations based on molecular simulations. *Chem. Phys. Lett.* **1994**, *222*, 529–539.

(S10) Lee, T.-S.; Lin, Z.; Allen, B. K.; Lin, C.; Radak, B. K.; Tao, Y.; Tsai, H.-C.; Sherman, W.; York, D. M. Improved Alchemical Free Energy Calculations with Optimized Smoothstep Softcore Potentials. *J. Chem. Theory Comput.* **2020**, *16*, 5512–5525.

(S11) Giese, T. J.; York, D. M. Variational Method for Networkwide Analysis of Relative Ligand Binding Free Energies with Loop Closure and Experimental Constraints. *J. Chem. Theory Comput.* **2021**, *17*, 1326–1336.

(S12) Kullback, S.; Leibler, R. A. On Information and Sufficiency. *Ann. Math. Statist.* **1951**, *22*, 79–86.

(S13) Zhang, S.; Giese, T. J.; Lee, T.-S.; York, D. M. Alchemical Enhanced Sampling with Optimized Phase Space Overlap. *J. Chem. Theory Comput.* **2024**, *20*, 3935–3953.

(S14) Nocedal, J. Updating quasi-Newton matrices with limited storage. *Math. Comput.* **1980**, *35*, 773–782.

(S15) Virtanen, P.; Gommers, R.; Oliphant, T. E.; Haberland, M.; Reddy, T.; Cournapeau, D.; Burovski, E.; Peterson, P.; Weckesser, W.; Bright, J.; van der Walt, S. J.; Brett, M.; Wilson, J.; Millman, K. J.; Mayorov, N.; Nelson, A. R. J.; Jones, E.; Kern, R.; Larson, E.; Carey, C. J.; Polat, I.; Feng, Y.; Moore, E. W.; VanderPlas, J.; Laxalde, D.; Perktold, J.; Cimrman, R.; Henriksen, I.; Quintero, E. A.; Harris, C. R.; Archibald, A. M.; Ribeiro, A. H.; Pedregosa, F.; van Mulbregt, P.; Vijaykumar, A.; Bardelli, A. P.; Rothberg, A.; Hilboll, A.; Kloeckner, A.; Scopatz, A.; Lee, A.; Rokem, A.; Woods, C. N.; Fulton, C.; Masson, C.; Häggström, C.; Fitzgerald, C.; Nicholson, D. A.; Hagen, D. R.; Pasechnik, D. V.; Olivetti, E.; Martin, E.; Wieser, E.; Silva, F.; Lenders, F.; Wilhelm, F.; Young, G.; Price, G. A.; Ingold, G.; Allen, G. E.; Lee, G. R.; Audren, H.; Probst, I.; Dietrich, J. P.; Silterra, J.; Webber, J. T.; Slavič, J.; Nothman, J.; Buchner, J.; Kulick, J.; Schönberger, J. L.; de Miranda Cardoso, J.; Reimer, J.; Harrington, J.; Rodríguez, J.; Nunez-Iglesias, J.; Kuczynski, J.; Tritz, K.; Thoma, M.; Newville, M.; Kümmerer, M.; Bolingbroke, M.; Tartre, M.;

Pak, M.; Smith, N. J.; Nowaczyk, N.; Shebanov, N.; Pavlyk, O.; Brodtkorb, P. A.; Lee, P.; McGibbon, R. T.; Feldbauer, R.; Lewis, S.; Tygier, S.; Sievert, S.; Vigna, S.; Peterson, S.; More, S.; Pudlik, T.; Oshima, T.; Pingel, T. J.; Robitaille, T. P.; Spura, T.; Jones, T. R.; Cera, T.; Leslie, T.; Zito, T.; Krauss, T.; Upadhyay, U.; Halchenko, Y. O.; Vázquez-Baeza, Y.; SciPy 1.0 Contributors, SciPy 1.0: fundamental algorithms for scientific computing in Python. *Nat. Methods* **2020**, *17*, 261–272.
